# 6-deoxy-6-amino chitosan: a preventative treatment in the tomato/*Botrytis cinerea* pathosystem

**DOI:** 10.3389/fpls.2023.1282050

**Published:** 2023-10-10

**Authors:** Naadirah Moola, Anwar Jardine, Kris Audenaert, Mohamed Suhail Rafudeen

**Affiliations:** ^1^ Laboratory of Plant Stress, Department of Molecular and Cell Biology, Faculty of Science, University of Cape Town, Cape Town, South Africa; ^2^ Department of Chemistry, Faculty of Science, University of Cape Town, Cape Town, South Africa; ^3^ Laboratory of Applied Mycology and Phenomics, Department of Plants and Crops, Faculty of Bioscience Engineering, Ghent University, Ghent, Belgium

**Keywords:** aminochitosan, Botrytis, tomato, priming, plant-pathogen interaction, antifungal activity, systemic resistance

## Abstract

6-deoxy-6-amino chitosan (aminochitosan) is a water-soluble chitosan derivative with an additional amine group at the C-6 position. This modification has improved aqueous solubility, *in vitro* antifungal activity and is hypothesized to have enhanced *in vivo* antifungal activity compared to native chitosan. Gray mold disease in tomatoes is caused by the fungus, *Botrytis cinerea*, and poses a severe threat both pre- and post-harvest. To investigate the optimal concentration of aminochitosan and its lower molecular weight fractions for antifungal and priming properties in the tomato/*B. cinerea* pathosystem, different concentrations of aminochitosan were tested *in vitro* on *B. cinerea* growth and sporulation and *in vivo* as a foliar pre-treatment in tomato leaves. The leaves were monitored for photosynthetic changes using multispectral imaging and hydrogen peroxide accumulation using DAB. Despite batch-to-batch variations in aminochitosan, it displayed significantly greater inhibition of *B. cinerea in vitro* than native chitosan at a minimum concentration of 1 mg/mL. A concentration-dependent increase in the *in vitro* antifungal activities was observed for radial growth, sporulation, and germination with maximum *in vitro* inhibition for all the biopolymer batches and lower MW fractions at 2.5 and 5 mg/mL, respectively. However, the inhibition threshold for aminochitosan was identified as 1 mg/mL for spores germinating *in vivo*, compared to the 2.5 mg/mL threshold *in vitro*. The pre-treatment of leaves displayed efficacy in priming direct and systemic resistance to *B. cinerea* infection at 4, 6 and 30 days post-inoculation by maintaining elevated F_v_/F_m_ activity and chlorophyll content due to a stronger and more rapid elicitation of the defense systems at earlier time points. Moreover, these defense systems appear to be ROS-independent at higher concentrations (1 and 2.5 mg/mL). In addition, aminochitosan accumulates in the cell membrane and therefore acts to increase the membrane permeability of cells after foliar spray. These observations corroborate the notion that aminochitosan biopolymers can exert their effects through both direct mechanisms of action and indirect immunostimulatory mechanisms. The contrast between *in vitro* and *in vivo* efficacy highlights the bimodal mechanisms of action of aminochitosan and the advantageous role of primed plant defense systems.

## 1 Introduction

Tomato (*Solanum lycopersicum* L.) is an important crop that alone accounted for almost 25% of the total global vegetable crop production increase between 2000 and 2021 (FAOSTAT, 2022). Gray mold disease is caused by the polyphagic, necrotrophic fungal pathogen, *Botrytis cinerea*, and affects over 1400 known hosts in 586 plant genera, including tomato (Fillinger and Elad, 2016). *B. cinerea* has a complex life cycle that results in varying symptoms across different plant tissues and organs (Fillinger and Elad, 2016; Poveda et al., 2020). It can infect all plant parts both pre- and post-harvest (including endophytic activity), and lie dormant or remain active during harvest or storage (Fillinger and Elad, 2016). Thus, economic impacts include direct losses (unmarketable crops or yield loss) and indirect losses (quality, harvest timing and control strategies) that amount to billions in annual economic losses (Fillinger and Elad, 2016; Poveda et al., 2020). To date, the predominant gray mold management strategy has been the use of fungicides, despite the challenges and concerns associated with its negative effects on the environment (Fenner et al., 2013) and human health (Verger and Boobis, 2013), lasting residues in food (Popp et al, 2013), and acquired fungicide resistance, resulting in an ever-increasing effective dosing requirement for crops (Pengfei Leng, 2011; Fillinger and Elad, 2016). These disadvantages have resulted in stricter regulations governing the application of fungicides and the permitted residue levels, resulting in a shift towards implementing eco-friendly alternatives (De Waard, 1993; Williamson et al., 2007). Biopolymers are thus viable alternatives to fungicides owing to their non-toxicity, multiple mechanisms of action (MOA), and broad-spectrum antimicrobial activity (Kaur et al., 2012).

Chitosan is a biopolymer of interest due to the above characteristics, with the addition of its biocompatibility, chemical versatility, and biodegradability properties (Kong et al., 2010; Verlee et al, 2017). Moreover, *B. cinerea* has been shown to be chitosan-sensitive due to the structural composition of its cell membrane (Palma-Guerrero et al., 2010). Produced via alkaline deacetylation of chitin; chitosan, and derivatives are biopolymers composed of *N*-acetyl glucosamine and glucosamine monomer units respectively (Verlee et al, 2017). The commercial sources of chitin used for chitosan synthesis are largely obtained from the crustacean exoskeletons derived from the waste of the seafood industry or are otherwise sourced from the exoskeletons of insects (Hadwiger, 2013; Liaqat and Eltem, 2018). However, chitosan’s insolubility in neutral aqueous solutions as well as moderate antimicrobial activity relative to chemical biocides have limited its commercial development in the agricultural sector (Hu et al., 2016; Romanazzi et al, 2018).

Various modifications of chitosan by means of *O*- or *N*-conjugation have been shown to improve physiochemical properties such as solubility and antimicrobial activity (Verlee et al, 2017; Brasselet et al., 2019). Some of the key factors differentiating the biological activity and solubility of chitosan derivatives are the presence of reactive amine group(s) and the ratio of amine to *N*-acetyl groups (Liaqat and Eltem, 2018; Poznanski et al, 2023). The reactive amine group(s) create a net positive charge and are proposed as integral to the mechanism of action. This net positive charge allows chitosan to interact with anionic surfaces via strong electrostatic interactions (Kong et al., 2010). Therefore, chitosan with an additional amine group termed 6-deoxy-6-amino chitosan (herein referred to as aminochitosan) was synthesized to improve its biological activity and water-solubility (Satoh et al., 2006).

Studies have reported that aminochitosan has improved transfection efficiency (Satoh et al., 2006) antibacterial activity (Yang et al., 2012) and antioxidant activity (Yang et al., 2015; Luan et al., 2018) when compared to native chitosan. However, due to the difficulties in dissolving aminochitosan in neutral pH water as prepared following the earlier methods stated, an improved synthesis of aminochitosan was deduced. Aminochitosan, soluble in water at pH 7, was synthesized in a shorter, greener, and more scalable synthetic protocol by Sayed et al. (2018). Compared to the abovementioned reported studies, this water-soluble aminochitosan is proposed to have improved biological activity and is soluble in water at a neutral pH (Sayed et al, 2018). However, other deterministic factors affecting the physiochemical properties and biological activity of chitosan include the degree of deacetylation (DDA), polymerization (DP), and substitution (DS), as well as the molecular weight (MW) (Bellich et al., 2016).

Chitosan’s biological activities are actioned through a triple-acting system of antimicrobial activities, film-forming properties, and the elicitation of plant defense systems (Xing et al., 2015; Romanazzi et al, 2018). The MOA and responses thereto vary depending on the pathosystem, microbial factors, physical state of chitosan, environmental factors, time of application, and intrinsic and extrinsic physiochemical properties of chitosan (El Hadrami et al., 2010; Kong et al., 2010; Poznanski et al, 2023). The MOAs for the antimicrobial activity are proposed to be through a combination of direct physiochemical interference with the pathogen, which includes the formation of a film layer, induction of pathogen-related morphological changes at all developmental stages, and direct interaction with DNA/chromatin (Rabea et al., 2003; Goy et al, 2009; Kong et al., 2010; Ana Niurka Hernández-Lauzardo, 2011; Hadwiger, 2013; Xing et al., 2015; Verlee et al, 2017; Luan et al., 2018; Romanazzi et al, 2018). In addition, the indirect MOA arises from the elicitation and exploitation of the plant’s innate immunity, resulting in induced resistance (IR) through various systemic mechanical, biochemical, and molecular changes within the plant (El Hadrami et al., 2010; Hadwiger, 2013; Aranega-Bou et al., 2014). IR can be elicited systemically through direct signal recognition in locally infected tissue or by priming, which may be activated by treatment with natural or synthetic chemicals like chitosan or through infection (Aranega-Bou et al., 2014; Mauch-Mani et al., 2017). Priming induces physiological, epigenetic, and metabolic changes upon the initial stimulus which is followed by a robust defense response that is faster and/or stronger upon subsequent exposures to stimuli with a generally low cost to plant fitness (Aranega-Bou et al., 2014; de Vega et al, 2018). Therefore, priming increases the capacity and efficiency of defense and resistance through amplified defense signals, rather than direct activation of defense responses (Aranega-Bou et al., 2014; Mauch-Mani et al., 2017).

As aminochitosan has not yet been investigated *in planta*, this is the first study to analyze its role as a protective priming agent in the tomato/*B. cinerea* pathosystem. Moreover, this is the first study to investigate the effects of aminochitosan and its batch-batch variability in synthesis as well as the bioactivity of lower MW fractions.

## 2 Materials and methods

### 2.1 Plant material

Tomato (*Solanum lycopersicum* L.*)* cv. Moneymaker seeds were collectively germinated before being transplanted into individual pots containing potting soil. Seedlings were grown at 23°C with an 8 hour light/16 hour dark cycle (Audenaert et al, 2002). After 5 weeks, when the plants consisted of tertiary leaves with five leaflets, 80-120 plants were randomized and used for each experiment.

### 2.2 Botrytis cinerea

*B. cinerea* isolate R16 (Faretra and Pollastro, 1991) was grown on potato dextrose agar (PDA) for 2 weeks at room temperature under 12 hour dark/12 hour UV light conditions. Control (mock) and spore suspensions were made, each containing 0.01 M glucose and 6.7 mM KH_2_PO_4_, with either 1 x 10^6^ spores/mL of *B. cinerea* (*B. cinerea* spore suspension) or distilled water (mock solution) added (Audenaert et al, 2002).

### 2.3 Biopolymers

Chitosan (CHT, crab origin, DDA > 90%) was purchased from AK Scientific Inc. 6-deoxy-6-amino chitosan (aminochitosan, shrimp shell origin, > 96% DDA, Sayed et al., 2018) with batch-to-batch variants termed diamino 1 (D1), diamino 2 (D2), and diamino 3 (D3), were synthesized by the Department of Chemistry at the University of Cape Town, South Africa. The batch-to-batch variants were approximately 15 kDa, as inferred from the 15 kDa dialysis MW cutoff used during purification. Additional fractionation was performed on the parent biopolymer (**Figure S1**), D3, with the following MW cut-offs: 3-5 kDa (Fraction 1, F1), 15 kDa (Fraction 2, F2), 20 kDa (Fraction 3, F3), 20-99 kDa (Fraction 4, F4) and 100 kDa (Fraction 5, F5). The biopolymers are henceforth termed either D1, D2, or D3 for the batch-to-batch variants and F1, F2, F3, F4 or F5 for the respective MW fractions. Biopolymer solutions were freshly prepared 1 day before the start of all experiments at the following concentrations: 0.5 mg/mL (0.05%), 1 mg/mL (0.1%), 2.5 mg/mL (0.25%), and 5 mg/mL (0.5%). Solutions for the aminochitosan biopolymers were prepared in distilled water, and chitosan was prepared in 1% (v/v) acetic acid. Working concentrations of chitosan had an acetic acid concentration of 0.1%. Biopolymer solutions were stirred overnight and sonicated for 2 hours before use.

### 2.4 Biopolymer application as foliar spray: direct and systemic

The biopolymers were assessed for two different MOA *in vivo*: the direct effects of biopolymer application, termed “direct treatment,” and the indirect, systemic effects of biopolymer application, termed “systemic treatment”. For both types of treatment, the tertiary leaves of 5-week-old tomato plants were pre-treated by foliar spray until run-off (approximately 1.4 mL per leaf) with the different concentrations of the biopolymers, 24 hours before *B. cinerea* inoculation (see section 2.3). For the direct treatment, all five leaflets were sprayed (**Figure S2A**). To assess the systemic effect, the first primary leaflet of each leaf was covered with foil before the remaining four leaflets were sprayed (**Figure S2A**).

### 2.5 Biopolymer elemental analysis

Elemental analyses of elemental composition ratios (carbon and nitrogen, C/N) and the degree of substitution for chitosan and the aminochitosan fractions (see section 2.3) were conducted on a Thermo Flash 1112 Series CHN Analyzer and the EA Euro 3000 by the Department of Chemistry at the University of Cape Town. The ratio C:N was used to determine the degree of substitution (DS) using the following equation (Sayed, 2018):


$$\text{DS}=\left[\frac{\left(\frac{\text{C}}{\text{N}}\text{derivative}\right)}{\left(\frac{\text{C}}{\text{N}}\text{chitosan}\right)}\right]\times \text{DDA}\left(96\%\right)$$

### 2.6 *In vitro*: antifungal assays

#### 2.6.1 Effects on mycelial radial inhibition

The direct effects of the biopolymers were assessed as in El-Ghaouth et al. (1992) using a mycelial radial growth assay (El-Ghaouth et al., 1992). 10 mm fungal discs taken from actively growing 2-week-old *B. cinerea* plates were placed centrally on PDA media amended with a biopolymer (CHT, D1, D2, D3, F1, F2, F3, or F5). The final concentrations of the amended media were 0.5, 1 or 2.5 mg/mL. Unamended PDA, water (PDA dilution control, data not shown), and 0.1% (v/v) acetic acid were used as controls. Plates were grown under 12 hour dark/12 hour UV (combined UVA and UVC) conditions for 11 days. Radial growth measurements (expressed as an average mycelial area in mm^2^) and macro-photos were taken at 1, 2, 3, 4, 5, 8, and 11 days post-initiation. The percentage inhibition of radial growth (PIRG%) was calculated as in El-Ghaouth et al., 1992. Experiments were performed with five biological replicates per treatment, per experiment, and repeated twice.

#### 2.6.2 Effects on sporulation

*B. cinerea* spores were harvested from the 11-day-old plates in 5 mL of water and filtered through sterile Miracloth (Pabón-Baquero et al., 2015). The concentration of spores was determined using a hemocytometer and expressed as average spore/mL. The experiment was repeated twice with 5 biological replicates for each biopolymer and concentration. The percentage inhibition of sporulation (PIS%) was calculated as in Al-Hetar et al., 2011 (Al-Hetar et al., 2011).

### 2.7 *In vivo*: direct and systemic effects in detached whole leaves and leaf discs

#### 2.7.1 Experiment set up, inoculation and lesion frequency

24 hours after spraying, whole leaves were excised at the base of the petiole before being wrapped in paper towels and placed on a tray. The leaves were then suspended above wet paper towels, with the stems immersed in distilled water. Individual leaflets were inoculated with two 10 µL droplets of either *B. cinerea* spore suspension or mock solution on either side of the midrib. The trays were then sealed with transparent lids to ensure a high-humidity environment and grown under a 16 hour light/8 hour dark cycle. Disease progression was assessed by counting the number of spreading necrotic lesions compared to resistant lesions (**Figure S2B**).

#### 2.7.2 Image analysis for phenotyping disease progression: F_v_/F_m_, ChlIdx, and mAriIdx

Leaflets were imaged using the CropReporter PathoViewer platform at 4 and 6 days post-inoculation (dpi). The non-sprayed first primary leaflet of each leaf was imaged to assess the systemic treatment effect in systemically sprayed leaves, while all five leaflets were imaged to assess the direct treatment. The PathoViewer (Department of Crops and Plants, Ghent University, Belgium), a non-invasive multispectral imaging platform, was used for the analysis of photosynthetic changes in real time, as in De Zutter et al. (2021). The platform used an automated, high-resolution, multispectral camera system mounted to a Cartesian-coordinate grid table contained in a light (Sun LED modules) chamber with controlled temperature and humidity (CropReporter, PhenoVation). The monochrome camera system captured absorption, reflection, and fluorescence patterns at a high temporal and spatial resolution of 6 µm and fitted with optical filters. The following parameters were calculated in a pixel-by-pixel manner from the obtained images: the average maximum efficiency of photosystem II (F_v_/F_m_) (Baker, 2008), RGB values, and the stress indices, namely the average chlorophyll fluorescence index (ChlIdx, a measure “leaf yellowing and chlorophyll content”) (De Zutter et al., 2021) and the average modified anthocyanin index (mAriIdx) (Gitelson et al, 2009). The PhenoVation imaging software and algorithms (PhenoVation, Wageningen, the Netherlands) were used to calculate the average F_v_/F_m_, ChlIdx, and mAriIdx along with the standard deviations for each leaflet from these images (De Zutter et al., 2021). As such, the effects of the biopolymers on the overall leaf health and disease progression were assessed based on the phenotypic changes observed over the course of the experiment (Baker, 2008; De Zutter et al., 2021).

#### 2.7.3 Time-trial analysis: hydrogen peroxide accumulation (DAB assay)

The protocols of Asselbergh et al. (2007) and Thordal-Christensen et al. (1997) were used with the following amendments: after spraying (see section 2.4) and 1 hour of drying, whole leaves were excised from multiple plants and randomized for each treatment. Leaf discs were taken with a 1 cm cork bore and floated (abaxial side down) in 24-well plates containing 1.5 mL of water per well. 24 hours after the leaves were sprayed, leaf discs were inoculated with two 5 µL droplets of mock or spore suspension on either side of the midrib (Audenaert et al, 2002; Asselbergh et al., 2007). The samples were allocated into different time groups, where the infection was allowed to establish for either 4, 8, 12, 24, 48, or 72 hours before staining. Prior to staining, the 24-well plates were imaged with the PathoViewer platform (see section 2.7.2) for macroscopic images. The protocol of Thordal-Christensen et al. (1997) was used for the 3’,3’-diaminobenzidine (DAB) staining and amended as follows: at each time point post-inoculation, the water was replaced with 1.5 mL of 1 mg/mL DAB. Leaf discs were floated for 4 hours before being de-stained (boiled) in a lactophenol mixture (phenol: glycerol: lactic acid: water: ethanol (1:1:1:1:2) for 30 minutes. Following H_2_O_2_ staining, fungal structures were stained with 0.02% (w/v) Trypan Blue in distilled water (for 30 sec). After staining, the leaf discs were mounted on glass slides in 50% (v/v) glycerol. Brightfield microscopy was performed with an Olympus BX-51 microscope and a Nikon Ti inverted Eclipse microscope using the NIS-Elements AR imaging software.

#### 2.7.4 Time trial analysis: spore germination

Leaf discs used in the DAB assay (see section 2.7.3) were used to analyze the effects of diamino 1 compared to water on spore germination at two time points, 16 hpi and 20 hours post inoculation (hpi).

#### 2.7.5 Time-trial analysis: gene expression of *SLACRE75*

This analysis was set up as the phenotyping experiment (see section 2.7.1) with the following amendments: Individual leaflets were harvested and considered biological replicates. Therefore, five biological replicates (five leaflets) were harvested from one tertiary leaf for the direct treatment and one biological replicate (one leaflet) for the systemic treatment. Leaflets were harvested and flash frozen at 6 and 9 hpi for the direct treatment and at 96 hpi for the systemic treatment (**Figure S2A**). The harvested tissue was analyzed for gene expression of *ACRE75*. Primers for *ACRE75* were synthesized using sequences from De Vega et al. (2021). The reference genes, *SICBL1* and *LSM7*, were selected from Rezzonico et al, (2018) and primers synthesized accordingly (Rezzonico, Nicot & Fahrentrapp, 2018). RNA was extracted using the PureLink^®^ Plant RNA Reagent (Thermo Fisher Scientific, Waltham, USA) as recommended. cDNA was synthesized from 1 µg of RNA using the Maxima First Strand cDNA Synthesis Kit with dsDNase (Thermo Fisher Scientific, Waltham, USA). RT-qPCR was conducted using KAPA SYBR^®^ FAST qPCR Master Mix (2X) Universal (KAPA Biosystems, Salt River, Cape Town) on a Rotor-Gene™ 6000 real-time rotary analyzer (Corbett Life Science, Sydney, Australia). The data was analyzed in qbase (Biogazelle, Zwijnaarde, Belgium) and normalized to the two reference genes. The maximum replicate variability was set to 0.1, and any replicate with a difference > 0.1 was excluded under quality control. The reference target stability levels were defined by thresholds set at 1 for the geNorm expression stability value and 0.3 for the coefficient of variation of the normalized reference gene relative quantities.

### 2.8 Statistical analysis

Plots were generated using the R software version 3.6.0 (R Core Team, 2020) and the packages ggplot2 (Wickham, 2016). The non-parametric Kruskal-Wallis test was used for multiple comparisons, followed by a *post hoc* analysis using Dunn’s test for pairwise comparisons. An FDR-corrected significance value of 0.05 was used for all analyses.

## 3 Results

### 3.1 *In vitro* antifungal activity of aminochitosan against *B. cinerea*

Two batches of aminochitosan were synthesized to assess the batch-to-batch variability and are referred to as diamino 1 (D1) and diamino 2 (D2). The biopolymers were assessed *in vitro* for their efficacy against *B. cinerea* compared to chitosan (CHT).

**Figure 1:**
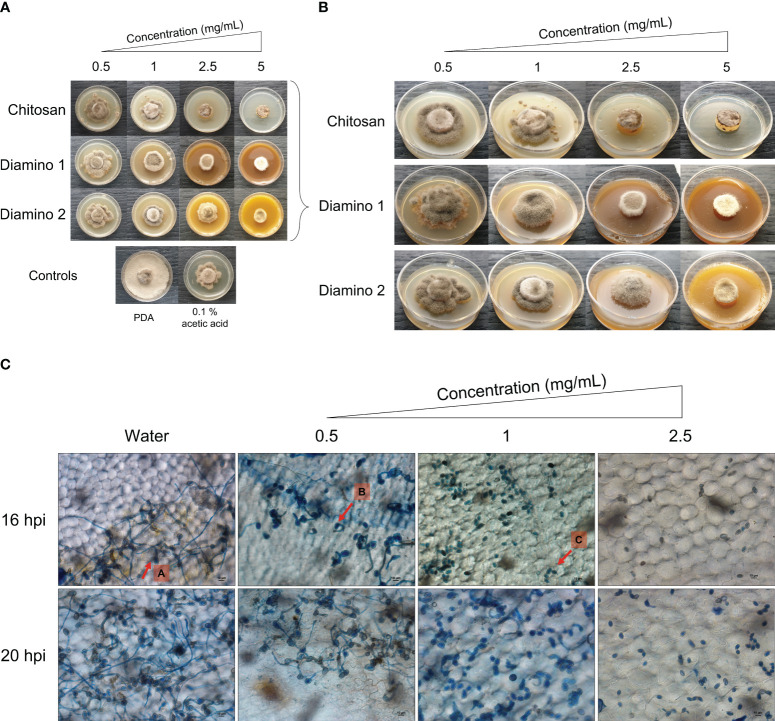
The direct antifungal effects of chitosan and aminochitosan batches on *B. cinerea* growth, 11 days after incubation. **(A)** An overview of the phenotypic effects relative to the controls (PDA and 0.1% (v/v) acetic acid). **(B)** A detailed view of the macroscopic and morphological changes. The images represent one of five biological replicates. The experiment was repeated twice. **(C)** The phenotypic effects of water and D1 at 0.5, 1 and 2.5 mg/mL concentrations on the germination, germ tube formation, and elongation of *B. cinerea* spores visualized at 40X magnification (scale bar = 10 µm.). The images display germination at 16 and 20 hours post inoculation. Arrows **(A-C)** indicate the average phenotype for each concentration. Spores were inoculated onto leaf discs and stained with Trypan Blue for visualization. The images represent the average of 4 biological replicates. The experiment was repeated once.

The biopolymer treatments displayed radial growth in concentric rings and excessively branched mycelia with a prevalence for “upward growth” (**Figure 1B**). In addition, the CHT treatments displayed haloes around the mycelial growth (data not shown). The direct *in vitro* antifungal activities of CHT, D1, and D2 showed a significant increase in inhibitory activity with increasing concentrations of the biopolymers compared to the PDA control (**Figures 1A, B**; **Table 1**). Notably, variations in the efficacy of the concentrations were observed across the biopolymers, as D1 exhibited significant radial growth inhibition (PIRG%) compared to CHT and D2 at 2.5 mg/mL (**Table 1**). To account for the inhibitory effects of acetic acid on fungal growth (data not shown), a 0.1% (v/v) acetic acid control was included as a control for CHT since CHT is only soluble in weak acids. The 0.1% acetic acid control was shown to be statistically different to the PDA control and generally no different to all biopolymer concentrations (**Table 1**). Maximum PIRG% for each of the biopolymers was observed between 2.5 and 5 mg/mL despite the large variance in the standard deviations (**Figure 1B**; **Table 1**).

**Table 1:** The effects of different concentrations of chitosan (CHT) and aminochitosan variants on the average mycelial radial inhibition and sporulation of *B. cinerea*, 11 days after incubation.

Treatment	Concentration (mg/mL)	Radial Inhibition		Sporulation	
		Growth area (mm^2^) ± SD	PIRG (%) ± SD	Spores/mL ± SD	PIS (%) ± SD
PDA	0	491 ± 0	0 ± 0^a^	21 133 ± 17 365	0 ± 0^a^
0.1% acetic acid	0	133 ± 39	73 ± 15^bc^	31 260 ± 17 442	7 ± 24^a^
Chitosan	0.5	298 ± 195	39 ± 32^b^	44 633 ± 33 383	-27 ± 56^a^
	1	70 ± 58	86 ± 10^cd^	35 967 ± 30 505	3 ± 57^a^
	2.5	56 ± 84	89 ± 14^de^	5 600 ± 5 808	84 ± 13^b^
	5	0 ± 0	100 ± 0^e^	100 ± 141	99 ± 1^b^
PDA	0	491 ± 0	0 ± 0^a^	21 133 ± 17 365	0 ± 0^a^
Diamino 1	0.5	279 ± 109	43 ± 16^b^	103 850 ± 120 703	-164 ± 217^b^
	1	118 ± 151	76 ± 22^bc^	16 200 ± 5 940	59 ± 15^ab^
	2.5	0 ± 0	100 ± 0^c^	0 ± 0	100 ± 0^c^
	5	0 ± 0	100 ± 0^c^	0 ± 0	100 ± 0^c^
PDA	0	491 ± 0	0 ± 0^a^	21 133 ± 17 365	0 ± 0^a^
Diamino 2	0.5	314 ± 148	36 ± 14^b^	65 400 ± 104 790	-70 ± 215^a^
	1	65 ± 27	87 ± 5^c^	33 600 ± 54 487	14 ± 138^a^
	2.5	34 ± 12	93 ± 2^cd^	1333 ± 14 045	97 ± 4^b^
	5	7 ± 11	98 ± 2^d^	450 ± 636	98 ± 3^b^

PIRG% = percentage inhibition of radial growth (PIRG), and PIS% = percentage inhibition of sporulation (PIS). A negative PIS% indicates growth greater than the control. Means ± SD (standard deviation) followed by the same superscript letter are not significantly different from each other (Kruskal-Wallis test followed by Dunn’s post-hoc test, p< 0.05). The values shown are the average of three experiments.

Intriguingly, the inhibitory effects of the biopolymers on the sporulation of *B. cinerea* displayed an increase in the number of spores/mL for CHT, D2, and D1 at 0.5 mg/mL compared to the PDA control which increased in that respective order (**Table 1**). This correlated with the phenotypic changes seen in the mycelial growth for the biopolymer treatments at 0.5 mg/mL. These were marked by the appearance of ashen, gray-colored masses in concentric rings compared to the PDA control, which displayed a uniformly light-colored growth (**Figures 1A, B**). Similarly, the 0.1% acetic acid control exhibited a comparable phenotypic effect on sporulation as 0.5 mg/mL of CHT (**Figure 1**). Overall, the biopolymers showed an initial increase in the average spores/mL at the lowest concentration assessed, followed by a decrease in the average spores/mL with increasing biopolymer concentrations (**Figures 1A, B**; **Table 1**).

As D1 exhibited greater radial growth inhibition compared to D2, it was selected to analyze the effects of aminochitosan on the germination of *B. cinerea in vivo.* The germination of spores on tomato leaflets sprayed with D1 showed increasing inhibition of germination and germ tube length with increasing concentrations of D1 (**Figure 1C**). Complete inhibition of germination can be observed at 2.5 mg/mL of D1 (**Figure 1C**). At concentrations of 0.5 and 1 mg/mL of D1, the germ tube lengths were shorter than the water treatment. Notably, 1 mg/mL of D1 demonstrated the greatest variability in both the number of spores germinating and the germ tube length (data not shown).

### 3.2 Multispectral analysis of the *in planta* direct and systemic effects of aminochitosan using F_v_/F_m_, chlorophyll index and anthocyanin index

To determine if aminochitosan exhibits comparable antifungal efficacy *in vivo* as the *in vitro* results, detached whole tomato leaves were pre-treated with aminochitosan 24 hours before *B. cinerea* inoculation (**Figure S2A**). Leaves were treated with one of the following variable combinations: the mode of application (direct/systemic), the treatment (biopolymer/water), and the inoculation solution (*B. cinerea*/mock). The disease progression of an artificial *B. cinerea* inoculation on tomato leaves (Benito et al., 1998) is displayed in **Figure S2B** while the induced resistance eliciting properties of aminochitosan are displayed in RGB images in **Figures S2A**, **S2B**.

A significant and completely resistant phenotype with 100% resistant lesions (i.e., no visible disease symptoms) was observed at 4 dpi for all concentrations of D1 applied as a direct treatment and was maintained at 6 dpi, with more than 95% resistant lesions for all concentrations assessed (**Figure S3C**; **Table 2**). These observations were compared to the water treatment + *B. cinerea* inoculation, of which 91 and 94% of lesions were necrotic at 4 and 6 dpi, respectively (**Table 2**). Direct treatment with D2 + *B. cinerea* inoculation also displayed significant resistance at 4 dpi and 6 dpi for all concentrations assessed but was less protective than D1 (**Figure S3C**; **Table 2**). CHT direct treatment + *B. cinerea* inoculated leaves had a lower efficacy at 4 and 6 dpi when compared to D1 and D2 treatment (**Table 2**; **Figure S3**). At 4 dpi, the 1 and 2.5 mg/mL concentrations of CHT treatment were significantly resistant. However, it is worth noting that the 0.1% acetic acid control + *B. cinerea* inoculated leaves displayed a small but nonsignificant increase in the percentage of resistant lesions (22%), compared to the water treatment (8%) and were not statistically different from the highest CHT concentration (**Figure S3C**; **Table 2**).

**Table 2:** The effects of chitosan (CHT), diamino 1 (D1), and diamino 2 (D2) on disease progression measured as the percentage of resistant lesions at 4 and 6 days post-inoculation (dpi).

Biopolymer	Concentration (mg/mL)	% Resistant lesions			
		Direct		Systemic	
		4 dpi	6 dpi	4 dpi	6 dpi
Water	0	8^a^	7^a^	17^a^	0^a^
0.1% acetic acid	0.1	21^ab^	13^a^	0^a^	0^a^
Chitosan	0.5	5^a^	2^a^	0^a^	0^a^
	1	68^c^	54^b^	0^a^	0^a^
	2.5	31^b^	10^a^	0^a^	0^a^
Water	0	9^a^	6^a^	0^a^	0^a^
Diamino 1	0.5	100^b^	96^b^	25^ab^	0^a^
	1	100^b^	95^b^	50^bc^	50^b^
	2.5	100^b^	98^b^	92^c^	58^b^
Water	0	44^a^	12^a^	28^a^	11^a^
Diamino 2	0.5	76^b^	23^a^	29^a^	14^a^
	1	58^c^	43^b^	42^a^	8^a^
	2.5	73^b^	55^b^	46^a^	19^a^

The data shows the average of two experiments, with 30-45 leaflets per experiment. The significance between concentrations for each polymer is denoted with letters. The same letters are not statistically different from each other (Kruskal-Wallis test followed by Dunn’s post-hoc test, p< 0.05).

The systemic protective effects of D1 treatment + *B. cinerea* inoculation at 4 dpi displayed significant resistant lesions at 50 and 90% for 1 and 2.5 mg/mL, respectively and were maintained at 6 dpi for 1 mg/mL (50%), with a decrease at 2.5 mg/mL (58%) (**Figure S3D**; **Table 2**). However, there were overlapping protective effects between the concentrations given the large standard deviations. In contrast to D1, D2’s protective effects at 4 and 6 dpi were nonsignificant when compared to the water treatment at 4 dpi but still maintained resistant lesions at 1 and 2.5 mg/mL with 42 and 46%, respectively (**Table 2**). CHT displayed the lowest efficacy of the biopolymers at both 4 and 6 dpi, with no resistant lesions at all concentrations tested.

**Figure 2:**
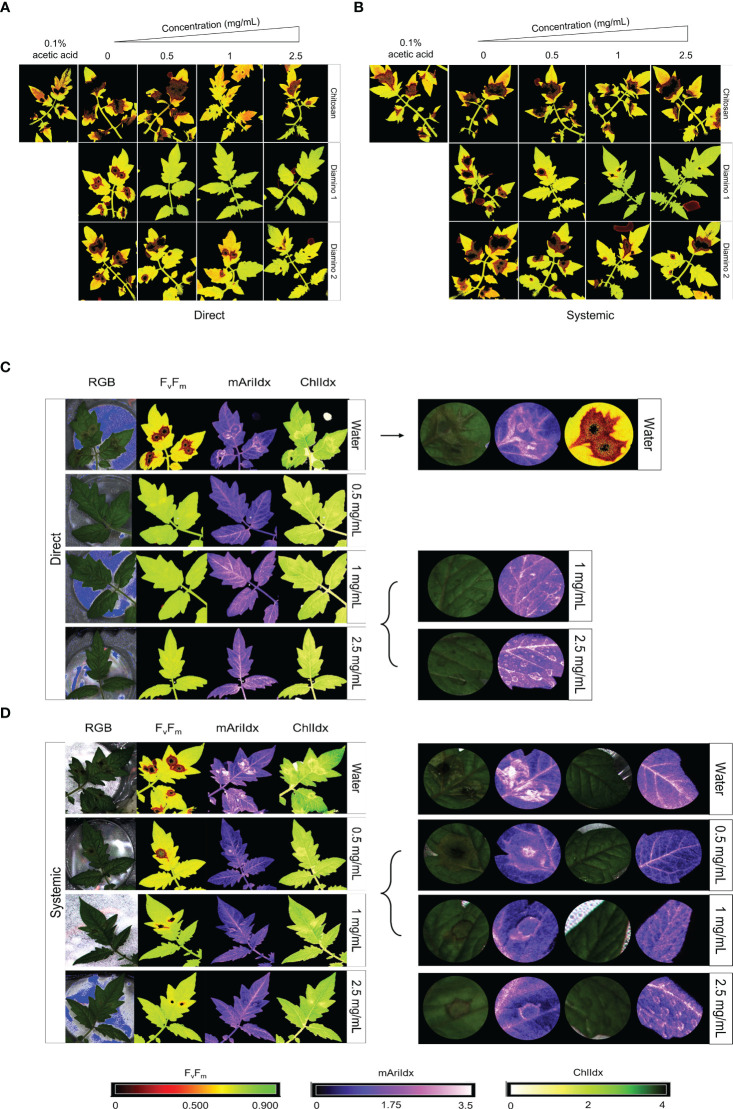
The phenotypic effects of chitosan (CHT), diamino 1 (D1), and diamino 2 (D2) treatment on the disease progression of *B. cinerea*, 4 days post-inoculation (dpi). The treatments were tested by direct or systemic application and imaged thereafter. Lesion development and progression were noted by the spreading of dark red/black (F_v_/F_m_, 0-0.5) or yellow/white (ChlIdx, 0-1.8) spots as measured by the false color scales. Healthy leaf tissue was noted as yellow/green (F_v_/F_m_,0.7-0.9) or green (ChlIdx, 1.9-2.5) by the false color scales. **(A)** Images displaying the direct effects of treatment on F_v_/F_m_ at 4 dpi. **(B)** Images displaying the systemic effects of treatment on F_v_/F_m_ at 4 dpi. **(C)** RGB, F_v_/F_m_, mAriIdx, and ChlIdx images of direct D1 treatment at 4 dpi and a detailed view of the effects with 1 and 2.5 mg/mL. **(D)** RGB, F_v_/F_m_, mAriIdx, and ChlIdx images of systemic D1 treatment at 4 dpi and a detailed view of all concentrations. Leaves were inoculated 24 hours after polymer spraying with two 10 µL droplets of a *B. cinerea* spore suspension (1 x 10^6^ spores/mL containing 0.01 M glucose and 6.7 mM KH_2_PO_4_). The images represent the average phenotype of two experiments.

**Figure 3:**
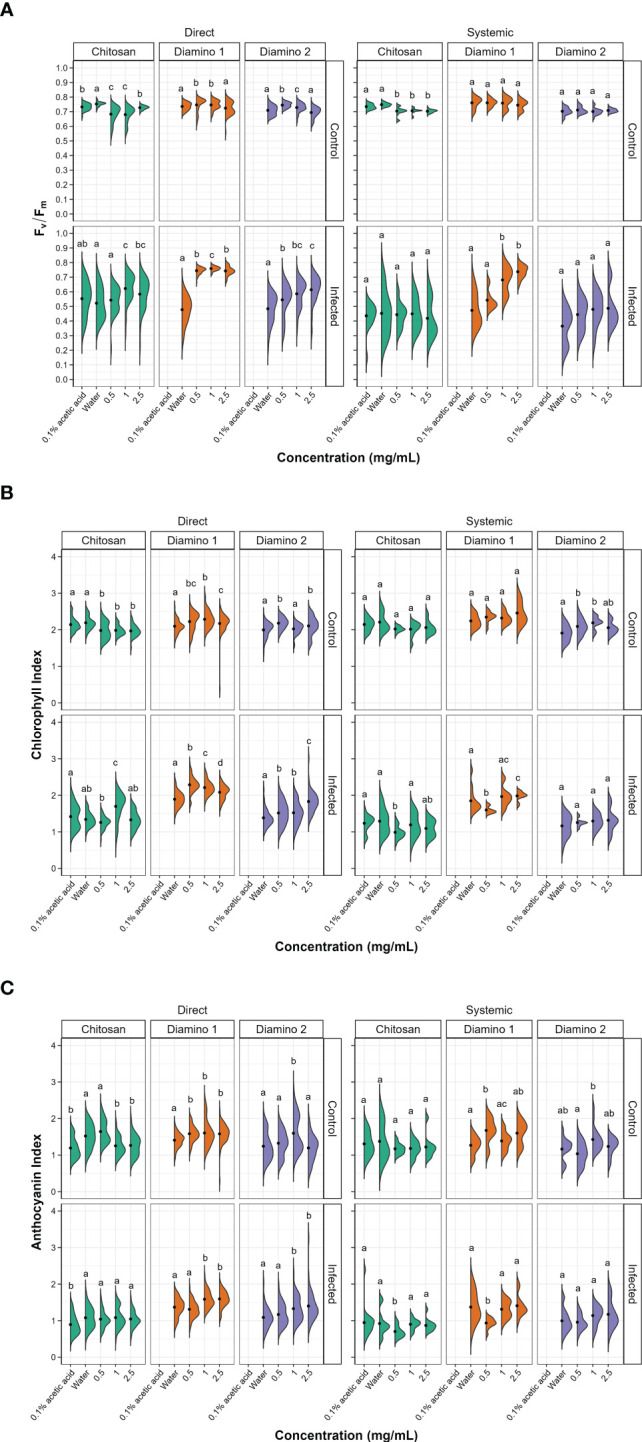
The effects of direct and systemic chitosan (CHT), diamino 1 (D1), diamino 2 (D2), and controls (water and 0.1% (v/v) acetic acid) treatment on the overall health of *B. cinerea* or mock inoculated leaves at 4 days post-inoculation. Overall health was assessed using **(A)** F_v_/F_m_, **(B)** chlorophyll index (ChlIdx) and **(C)** the modified anthocyanin index (mAriIdx). n = 45 leaflets per treatment. The black dots represent the mean of each half violin. Concentrations with different letters are statistically significant (Kruskal-Wallis test followed by Dunn’s *post-hoc* test, p< 0.05).

Images quantifying the changes in the photosynthetic performance of leaves treated and/or inoculated were used to assess the health of the leaf and/or disease progression of *B. cinerea* inoculation at 4 dpi. The photosynthetic performance was measured by quantifying the efficiency of photosystem II (F_v_/F_m_) and the stress indices, namely the chlorophyll index (ChlIdx) and the modified anthocyanin index (mAriIdx). **Figures 2A, B**, **S3** display images that visualize the effects of the direct and systemic biopolymer treatment on lesion development. **Figures 3**, **S4** show the distributions and mean values for F_v_/F_m_ (**Figures 3A**, **S3**), ChlIdx (**Figure 3B**), and mAriIdx (**Figure 3C**). All observations were compared to the water treatment + *B. cinerea* inoculated images (**Figure 2** **)** and the distributions (**Figures 3A**, **S4**) at 4 dpi.

#### 3.2.1 Direct biopolymer or water treatment and *B. cinerea* inoculation

The direct application of D1 treatment + *B. cinerea* inoculation resulted in significant F_v_/F_m_ values that were consistently higher than the F_v_/F_m_ values for D2 and CHT at all concentrations (**Figures 3A**, **S3A**). This was visualized by the absence of red lesions in the F_v_/F_m_ images and correlated with the F_v_/F_m_ distributions (**Figures 2A, C**). The direct treatment with D2 + *B. cinerea* inoculation also resulted in significant average F_v_/F_m_ values at all concentrations, but with a concentration dependent increase (**Figure 3A** **)**. The direct treatment with CHT + *B. cinerea* inoculation significantly increased the average F_v_/F_m_ values for 1 and 2.5 mg/mL concentrations, with 0.1% acetic acid having the same significance as the 2.5 mg/mL concentration (**Figures 3A**, **S3A**). The significant protective effects *in vitro* (**Table 1**) and increased protective effects *in vivo* (**Table 2**) for 0.1% acetic acid were also noted phenotypically by the reduced red lesion sizes (**Figure 2A**) and in the increase in the distribution and average F_v_/F_m_ values when compared to the water treatment (**Figures 3A**, **S3A**). Correspondingly, direct 0.1% acetic acid treatment + *B. cinerea* inoculation resulted in a similar nonsignificant increase in the average ChlIdx values (**Figure 3B**). Direct D1 and D2 treatment + *B. cinerea* inoculation showed a significant increase in the distribution of ChlIdx values at all concentrations assessed, with notable differences between the concentrations of D1 treatment (**Figure 3B**).

The mAriIdx values for direct treatment with CHT + *B. cinerea* inoculation were the same as for the water treatment (**Figure 3C**). However, the 0.1% acetic acid control was statistically lower than the water treatment and all concentrations of CHT (**Figure 3C**). In contrast, both D1 and D2 direct treatment + *B. cinerea inoculation* had statistical increases in the average mAriIdx at 1 and 2.5 mg/mL concentrations (**Figure 3C**). This was noted phenotypically in **Figure 2C**, where higher levels of mAriIdx are visible at the sites corresponding to 1 and 2.5 mg/mL of D1 treatment. Visually, this appeared concentration-dependent, as the accumulation was more visible at 2.5 mg/mL compared to 1 mg/mL and was not observed at 0.5 mg/mL.

#### 3.2.2 Systemic biopolymer or water treatment and *B. cinerea* inoculation

The F_v_/F_m_ values for the systemic D1 treated + *B. cinerea* inoculated leaflets corroborated the phenotyping data (**Figure 2B**) and were also significantly higher than the water treatment (**Figures 3A**, **S3B**) with a concentration-dependent increase in the average F_v_/F_m_ (**Figure 3A**). D1 also displayed marked differences in the proportion of healthy F_v_/F_m_ levels, 4 and 5, at all concentrations assessed compared to D2 and CHT (**Figure S4B**). Although the F_v_/F_m_ distributions of D2 did not exhibit a significant difference from the water treatment, the data points tended to cluster at higher values compared to the water treatment. This suggests that some protective effects may have been elicited (**Figure 3A**). Like the phenotyping data in **Figure 2B** and section 3.2, the average F_v_/F_m_ values for CHT, and 0.1% acetic acid treated + *B. cinerea* inoculated leaflets were nonsignificant when compared to the water treatment (**Figures 2B**, **3A**, **S3B**). The distribution of ChlIdx values for the D1, D2 and CHT systemically treated + *B. cinerea* inoculated leaflets was nonsignificant at all concentrations assessed except for 0.5 mg/mL of the D1 and CHT treatments, which were lower than the water treatment (**Figure 3B**).

Correspondingly, a significant decrease in the average mAriIdx at 0.5 mg/mL compared to the water treatment for D1 and CHT systemically treated + *B. cinerea* inoculated leaflets was also seen (**Figure 3C**). However, in contrast to the ChlIdx, a nonsignificant increase in mAriIdx distribution was observed at 1 and 2.5 mg/mL for D1 and D2 systemically treated + *B. cinerea* inoculated leaflets (**Figure 3C**). This was noted phenotypically in **Figure 2D**, where higher levels of anthocyanin were visible at the sites corresponding to 1 and 2.5 mg/mL of D1 treatment on systemically treated leaves.

#### 3.2.3 Direct biopolymer or water treatment and mock inoculation

D1 and D2 directly treated and mock inoculated leaflets displayed significant increases in the average F_v_/F_m_ values for 0.5 and 1 mg/mL, with a nonsignificant decrease in the average for the 2.5 mg/mL concentration (**Figure S4A**). A similar increase was observed for the average ChlIdx values for D1 and D2 directly treated and mock inoculated leaflets (**Figure 3B**). D1 treatment showed significant increases at all concentrations, whereas D2 treatment was only significant at 0.5 and 2.5 mg/mL (**Figure 3B**). D1 and D2 treatment displayed a significant increase in the average mAriIdx values for all concentrations, while D2 was only significant at 1 mg/mL (**Figure 3C**). This increase was visible in the D1 phenotyping images in **Figure S3C**, where areas with residual dry droplets correspond to higher mAriIdx values (according to the false color scale).

Contrastingly, for leaflets directly treated with CHT and mock inoculated, a significant decrease in the average F_v_/F_m_, ChlIdx, and mAriIdx values was observed at all concentrations assessed (except 0.5 mg/mL mAriIdx) (**Figures 3A–C**). This significant decrease in F_v_/F_m_ and mAriIdx was also observed for the 0.1% acetic acid treatment + *B. cinerea* inoculated leaves when compared to the water treatment + *B. cinerea* inoculated leaves (**Figures 3A–C**, **S3A** **)**.

#### 3.2.4 Systemic biopolymer or water treatment and mock inoculation

For the systemic application of D1 and D2 treatments and mock inoculation, no differences were seen in the distribution or average F_v_/F_m_ values for all concentrations assessed compared to the water treatment (**Figure 3A**). However, when looking at the distribution of the F_v_/F_m_ levels in **Figure S4B**, D1 displayed a higher proportion of levels 4 and 5 compared to D2 (**Figure S4B**). D1 systemically treated and mock inoculated leaflets displayed a nonsignificant increase in ChlIdx values at all concentrations, whereas D2 treated and mock inoculated leaflets were significantly greater at 0.5 and 1 mg/mL concentrations (**Figure 3B**). The average mAriIdx values for D1 and D2 treatments were not significant as the distributions were large, often with two clusters of data points indicating protective effects in a fraction of the leaflets assessed (**Figure 3C**). This increase was visible in the D1 phenotyping images in **Figure S3D**. Additionally, as in the systemic *B. cinerea* inoculated leaflets, little to no anthocyanin accumulated at the site of infection when treated with 1 and 2.5 mg/mL of D1 but appeared phenotypically similar at 0.5 mg/mL (**Figure 2C**). Treatment with CHT and mock inoculation was significantly lower at all concentrations for the average F_v_/F_m_ (**Figures 3A**, **S3B**). There were no changes observed in the ChlIdx and mAriIdx values at all concentrations of CHT treatment and mock inoculation assessed (**Figures 3B, C**).

### 3.3 Characterizing an early defense response in tomato leaflets: aminochitosan and H_2_O_2_ production

The production of reactive oxygen species (ROS) is frequently observed as a dominant and early defense response (Thordal-Christensen et al., 1997). Hence the impact of D1 on hydrogen peroxide (H_2_O_2_) production was evaluated in a time course series using DAB staining to compare H_2_O_2_ accumulation at the site of inoculation. This method yields brown precipitates that indicate the presence of H_2_O_2_ accumulation allowing both macroscopic and microscopic assessment.

#### 3.3.1 Macroscopic observations

**Figure 4:**
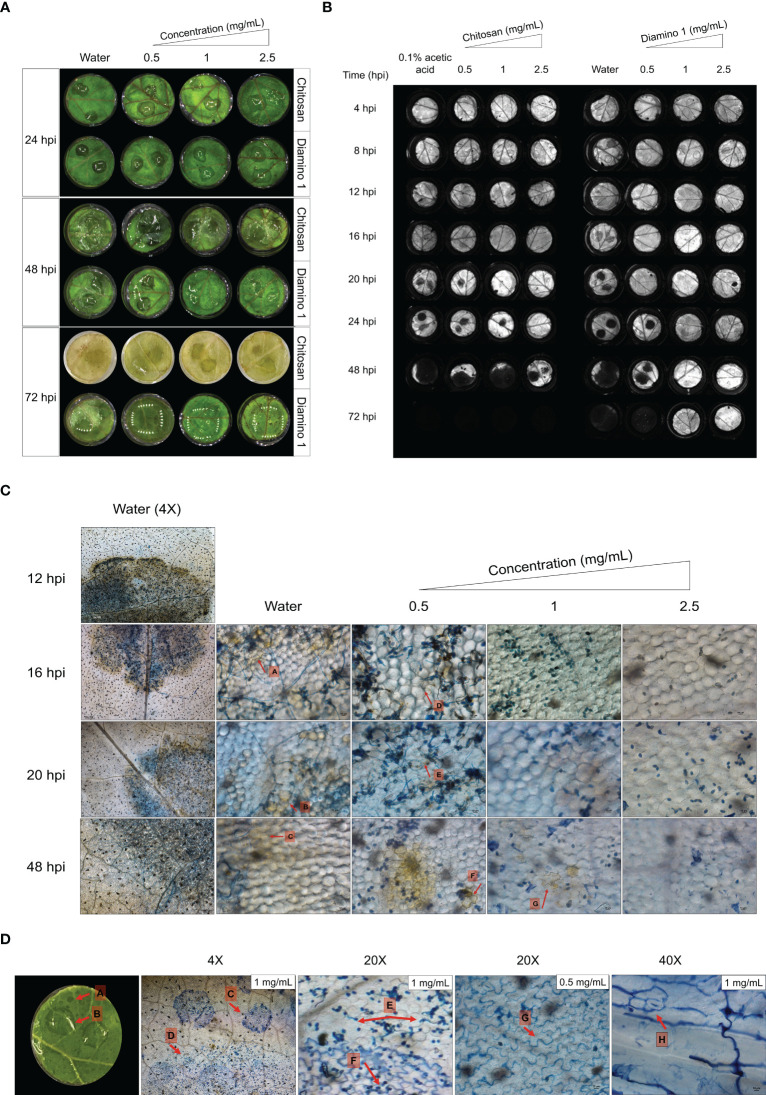
The temporal progression of *B. cinerea* disease symptoms on leaf discs treated with chitosan (CHT) and diamino 1 (D1). **(A)** The macroscopic development of spreading lesions over 24, 48 and 72 hpi. Lesions are denoted by their signature “wet” and “brown” phenotype at 48 hpi, followed by the growth of white mycelia at 72 hpi. **(B)** A chlorophyll fluorescence image showing the temporal development of lesions highlighted by the absence of chlorophyll (dark lesions) at the inoculation sites for the different time points. One of five biological replicates are shown. **(C)** DAB staining visualizing the accumulation or absence of H_2_O_2_ at 4X (water only) and 20X (water and D1 treatment). The experiment was repeated twice. **(D)** The co-staining of D1 with Trypan Blue. The accumulation of D1 in the walls of cells as indicated by arrows G and H. The diamino droplets sprayed onto the leaflets are labeled with arrows, A-C. Differences in the germination of spores covered by visible droplets compared to free spores are indicated by arrows C-F. The images represent the average of 4 biological replicates. The experiment was repeated once. Scale bar 4X magnification = 200 µm, 20X magnification = 100 µm and 40X magnification = 10 µm.

The macroscopic progression of disease symptoms were visualized over time using RGB and dark-adapted chlorophyll fluorescence (F_v_/F_m_) images (**Figures 4A**, **B** respectively) as the use of chlorophyll fluorescence allowed for earlier detection of disease symptoms (Pavicic et al., 2021).

In the RGB images, disease symptoms were only observable from 48 hpi for both CHT and D1 (**Figure 4A**). For the chlorophyll fluorescence images, dark spots on the leaflets that signify the lack of chlorophyll fluorescence served as an indicator for necrotic lesions. Mock inoculated leaflets displayed no dark spots (data not shown). The initial development of necrotic lesions was first observed at 16 hpi for the water treatment, at 20 hpi for 0.05 mg/mL of CHT treatment and at 24 hpi for 0.05 mg/mL of D1 treatment (**Figure 4B**). D1 treatment significantly protected against necrotic lesion development for 1 and 2.5 mg/mL up to and including 72 hpi (**Figure 4A**; **Table S1**). Lesion development for 0.1% acetic acid was protective up to 20 hpi compared to the water treatment at 16 hpi (**Figure 4A**; **Table S1**).

#### 3.3.2 Microscopic observations

No H_2_O_2_ accumulation was observed in the water/biopolymer treated and mock inoculated leaflets (data not shown). In the water treated + *B. cinerea* inoculated leaflets, H_2_O_2_ accumulation was visible at 4X magnification around the entire lesion perimeter and within the infection droplet from 12 hpi (**Figure 4C**). A decrease in the intensity of DAB staining was observed for the water treated leaflets between 24 and 48 hpi (**Figure 4C**).

Intriguingly, leaflets treated with D1 displayed a decrease in H_2_O_2_ accumulation with an increase in concentration as well as an increase in the intensity of DAB staining over time (**Figure 4C**). Therefore, the time taken to accumulate H_2_O_2_ levels comparable to the water treated leaflets was only achieved at later time points. Leaflets treated with 0.5 mg/mL of D1 displayed lesions with sparse areas of minimally visible H_2_O_2_ accumulation at 16 and 20 hpi (**Figure 4C** **, arrows D** and **E**) with a minimal increase in the intensity of DAB staining at 24 hpi (**Figure 4C** **, arrow F)**. For leaflets treated with 1 and 2.5 mg/mL of D1, no H_2_O_2_ accumulation was visible up to 16 hpi and 20 hpi (for 2.5 mg/mL) (**Figure 4C**). Between 20 and 48 hpi, D1 at 1 mg/mL displayed a low intensity of DAB staining in few cells (**Figure 4C** **, arrow G**) with D1 at 2.5 mg/mL only displaying H_2_O_2_ accumulation observed at 48 hpi (**Figure 4C** **)**.

The microscopic observations revealed an interaction between D1 and Trypan Blue (**Figure 4D** **, arrows C**). The area occupied by the droplets corresponded with the D1 droplet residues that were separate to the *B. cinerea* droplet residue (**Figure 4D** **, arrow A** and **B,** respectively). At higher magnification, the droplet areas also displayed an accumulation of Trypan Blue within the anticlinal walls of cells within the epidermis of the leaf tissue and exhibits the same lobed shape as the cells (**Figure 4D** **, arrow G** and **H**). Most notably, the spores beneath the D1 droplet area have little to no germinated spores when compared to the spores within the inoculation droplet that do not intersect with the D1 droplet (**Figure 4D** **, arrows E** and **F**).

### 3.3 The *in vitro* and *in vivo* efficacy of molecular weight variants of aminochitosan

Due to the observable differences in the *in vitro* and *in vivo* efficacy of D1 and D2, a third biopolymer batch was synthesized and further fractionated to allow for chemical and biological characterization of the different MW fractions. The third biopolymer batch will herein be referred to as diamino 3 (D3), and the D3 MW fractions will be referred to as fractions 1-5 (F1-F5).

#### 3.3.1 Elemental analysis of D3 and D3 lower MW fractions

Elemental analysis (EA) was used to determine whether varying efficacies of the aminochitosan batches were due to differences in their nitrogen composition. EA was conducted by identifying the percentage of carbon (C), nitrogen (N) and hydrogen (H) in D1, D2, D3, and the D3 MW fractions, F1 (3.5–5 kDa), F2 (15 kDa), F3 (20 kDa), F4 (20–99 kDa), and F5 (100 kDa). Data including the percentage of hydrogen and sulfur are not shown. The EA data displayed a clear increase in the percentage of nitrogen content for D1, D2, D3, F1, F2, F3, and F5 compared to native CHT (**Table 3**). F4 displayed the lowest percentage of nitrogen compared to the MW fractions and was also lower than the nitrogen content for CHT. In addition to determining the elemental composition, the ratio of carbon to nitrogen (calculated as C/N) was used to determine the degree of substitution (DS), the number of hydroxyl groups substituted with amino groups; a key factor when evaluating the formation of aminochitosan (Sayed, 2018). The DS values for D1 and D1 were within 0.08 of each other and were therefore within close range. The lowest DS was obtained for D3 (0.63), while the highest DS was obtained for F4 (0.81) (**Table 3**). D1 (0.76) F2 (0.77), F3 (0.78) and F5 (0.78) displayed the most similar DS (**Table 3**).

**Table 3:** Elemental analysis of carbon (C) and nitrogen (N) for chitosan (CHT), diamino 1 (D1), diamino 2 (D2), diamino 3 (D3), and diamino 3 MW fractions (F1-F5).

Polymers			C/N	DS
	*C (%)	*N (%)		
Chitosan	44.04	6.62	6.62	–
Diamino 1	44.24	9.40	4.70	0.76
Diamino 2	38.14	7.94	4.80	0.70
Diamino 3	44.15	10.10	4.37	0.63
3.5 - 5 kDa (F1)	35.43	6.83	5.19	0.75
15 kDa (F2)	37.03	6.98	5.31	0.77
20 kDa (F3)	40.88	7.64	5.35	0.78
20 - 99 kDa (F4)	31.49	5.67	5.55	0.81
100 kDa (F5)	39.86	7.45	5.35	0.78

* = as determined by elemental analysis. C/N = the ratio of carbon to nitrogen used to calculate the DS. DS = degree of substitution which denotes the number of -OH groups that were substituted with amine groups. The data represent the average of two experiments.

#### 3.3.2 The *in vitro* effects of different aminochitosan MW fractions

**Figure 5:**
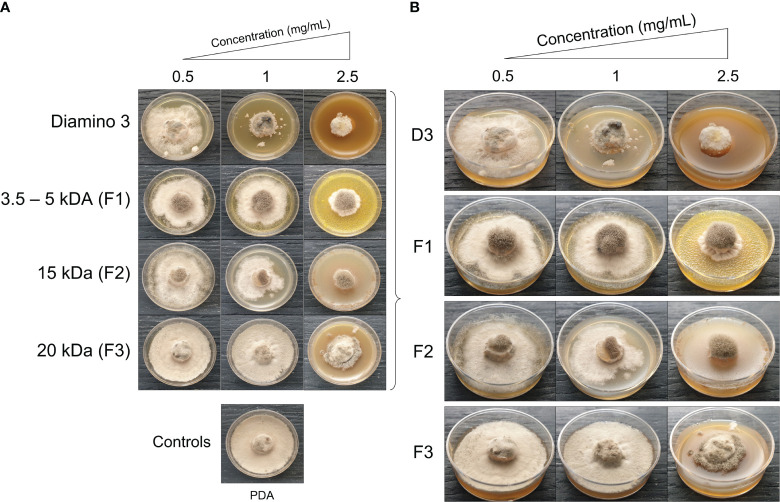
The phenotypic effects of diamino 3 (D3) and the D3 lower MW fractions (F1-F3) on the radial growth *of B. cinerea*, 11 days after incubation. **(A)** An overview of the effects relative to the PDA control. **(B)** A detailed view of the macroscopic and morphological changes. The images represent one of five biological replicates. The experiments were repeated twice.

The antifungal activity of D3 and the D3 MW fractions was investigated, with the quantitative and phenotypic effects shown in **Table 4** and **Figure 5**, respectively. A statistical increase in the PIRG% was observed for D3 treatment at 1 mg/mL and 2.5 mg/mL when compared to the PDA control (**Table 4**). Furthermore, a statistical increase in the PIS% was observed for all concentrations of D3 when compared to the PDA control (**Table 4**).

**Table 4:** The effects of various concentrations of diamino 3 (D3) and the D3 MW fractions (F1-F3) on the average mycelial radial growth and sporulation of *B. cinerea*, 11 days after incubation.

Treatment	Concentration (mg/mL)	Radial Inhibition		Sporulation	
		Growth area (mm^2^) ± SD	PIRG (%) ± SD	Spores/mL ± SD	PIS (%) ± SD
PDA	0	491 ± 0	0 ± 0^a^	21 133 ± 17 365	0 ± 0^a^
Diamino 3	0.5	457 ± 48	7 ± 7^a^	5 500 ± 1 980	77 ± 18^b^
	1	237 ± 259	52 ± 37^b^	4 750 ± 4 455	86 ± 8^b^
	2.5	49 ± 58	90 ± 8^c^	5 850 ± 8 132	85 ± 21^b^
	5	20 ± 33	96 ± 0^c^	300 ± 0	100 ± 0^b^
PDA	0	491 ± 0	0.00^a^	11 900 ± 14 100	0^a^
F1 (3.5-5 kDa)	0.5	358 ± 87	28.36^ab^	8 300 ± 4 600	30.25^a^
	1	235 ± 93	52.23^bc^	3 700 ± 4 200	68.91^ab^
	2.5	47 ± 38	90.49^c^	500 ± 0	95.80^b^
PDA	0	491 ± 0	0.00^a^	11 900 ± 14 100	0^a^
F2 (15 kDa)	0.5	373 ± 162	23.97^ab^	23 500 ± 32 200	-97.48^a^
	1	270 ± 133	45.01^bc^	2000 ± 3 600	83.19^b^
	2.5	0 ± 0	100.00^c^	100 ± 200	99.16^b^
PDA	0	491 ± 0	0.00^a^	11 900 ± 14 100	0^ab^
F3 (20 kDa)	0.5	455 ± 80	7.33^a^	5 500 ± 5 500	53.78^ac^
	1	491 ± 0	0.03^a^	1 500 ± 900	87.39^c^
	2.5	121 ± 75	75.42^b^	19 100 ± 9 100	-60.50^b^

PIRG% = percentage inhibition of radial growth (PIRG), and PIS% = percentage inhibition of sporulation (PIS). A negative PIS% indicates growth greater than the control. Means ± SD (standard deviation) followed by the same superscript letter are not significantly different from each other (Kruskal-Wallis test followed by Dunn’s post-hoc test, p< 0.05). The values shown are the average of three experiments for D3 and two experiments for the fractions.

When comparing the phenotype and radial inhibition of the lower MW fractions, F1 and F2 appear to perform better than D3, while F1 and F3 appear to perform similarly to D3, at 1 and 0.5 mg/mL respectively (**Figure 5**; **Table 4**). No significant differences in the efficacy between F1, F2, and D3 on the phenotype and radial inhibition were observed at 1 mg/mL, while F3 was significantly different. In addition, F1, F2, and D3 were similar at 2.5 mg/mL whereas the efficacy of F3 at 2.5 mg/mL was significantly lower at the same concentration (**Table 4**; **Figure 5**).

When compared to the PDA control, F1 and F2 showed significant inhibitory effects on sporulation at 1 and 2.5 mg/mL, and at 1 mg/mL for F3 (**Table 4**). Large standard deviations for the sporulation data are to be noted as limiting factors. F5 displayed no effects on radial inhibition and sporulation at all concentrations assessed when compared to the PDA control (data not shown).

#### 3.3.3 The *in vivo* effects of aminochitosan 3 (D3) and the D3 MW fractions, on eliciting resistance in the tomato/*B. cinerea* pathosystem

**Figure 6:**
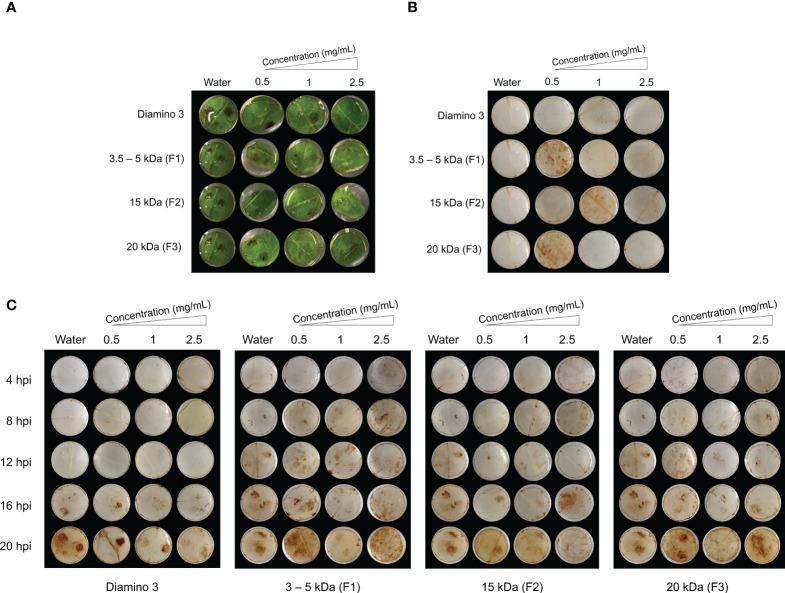
The temporal accumulation of H_2_O_2_ in leaf discs treated with diamino 3 (D3) and D3 lower MW fractions (F1-F3) and visualized with DAB staining at 4, 8, 12, 16 and 20 hours post-inoculation (hpi) with *B. cinerea*. **(A)** Macroscopic images of DAB staining at 20 hpi displaying the progression of disease symptoms as noted by the appearance of brown spots in the RGB image. **(B)** Macroscopic image of DAB staining in de-stained leaf discs sprayed with different polymer concentrations and inoculated with a mock solution at 20 hpi. **(C)** The macroscopic, temporal H_2_O_2_ accumulation at 4, 8, 12, 16, and 20 hpi. This image correlates with the RGB image in **(A)**. Leaf discs were inoculated with two 10 µL droplets of a *B. cinerea* spore solution (1 x 10^6^ spores/mL containing 0.01 M glucose and 6.7 mM KH_2_PO_4_) 24 hours after spraying. The image here represents one of four biological replicates.

The direct and systemic effects of D3 and the D3 lower MW fractions were analyzed for their efficacy in eliciting a resistant phenotype at 4, 6, and 30 dpi (**Figure S5**; **Table 5**) and H_2_O_2_ production at 4, 8, 12,16, and 20 hpi (**Figure 6**). When compared to the water treatment, direct treatment with D3, F2, and F3 significantly increased the percentage of resistant lesions at 4 and 6 dpi (**Table 5**; **Figure S5**). Notably, at 4 and 6 dpi, F2 and F3 displayed a decrease in the percentage of resistant lesions at 2.5 mg/mL when compared to 1 mg/mL of the respective biopolymer (**Table 5**). Direct treatment with F1 statistically increased the resistant phenotype at 1 and 2.5 mg/mL at both 4 and 6 dpi compared to the water treatment but was less effective than D3 (except at 6 dpi for 2.5 mg/mL), F2 and F3 (**Table 5**).

**Table 5:** The effects of various concentrations of diamino 3 (D3) and the D3 MW fractions (F1-F3) on disease progression measured as the percentage of resistant lesions at 4 and 6 days post-inoculation (dpi).

Biopolymer	Concentration (mg/mL)	% Resistant lesions			
		Direct		Systemic	
		4 dpi	6 dpi	4 dpi	6 dpi
Water	0	36^a^	21^a^	37^a^	17^a^
Diamino 3	0.5	63^b^	42^b^	57^ab^	27^ab^
	1	80^c^	63^c^	56^ab^	47^bc^
	2.5	83^c^	56^c^	75^b^	64^c^
Water	0	34^a^	28^a^	38^a^	29^a^
F1 (3.5-5 kDa)	0.5	47^ab^	43^ab^	67^b^	58^bc^
	1	59^b^	47^b^	33^ab^	17^ab^
	2.5	79^c^	69^c^	83^b^	67^c^
Water	0	34^a^	29^a^	38^a^	29^a^
F2 (15 kDa)	0.5	100^b^	99^b^	67^b^	50^a^
	1	99^b^	97^b^	83^b^	75^c^
	2.5	79^c^	79^c^	75^b^	67^b^
Water	0	34^a^	28^a^	38^a^	29^a^
F3 (20 kDa)	0.5	98^b^	88^b^	50^ab^	50^ab^
	1	96^b^	96^b^	100^b^	100^b^
	2.5	86^b^	81^b^	67^b^	50^ab^

The data shows the average of two experiments, with 30-90 leaflets per experiment. The significance between concentrations for each polymer is denoted with superscript letters. The same letters are not statistically different from each other (Kruskal-Wallis test followed by Dunn’s post-hoc test, p< 0.05).

The systemic treatment yielded variable results at the different concentrations applied due to large standard deviations with overlapping ranges (**Table 5**). Systemic treatment with D3, F2, and F3 was significantly protective at 1 and 2.5 mg/mL when compared to the water treatment at 4 and 6 dpi (**Table 5**). Systemic F1 treatment, when compared to the water treatment, was significantly protective at 0.5 and 2.5 mg/mL at 4 and 6 dpi (**Table 5**). Furthermore, the systemic protection provided by F2 was greater than D3 (**Table 5**). Notably, similar to the results from direct treatment, a decrease in the percentage of resistant lesions was observed for 2.5 mg/mL of D3, F2, and F3 treatments at 4 and 6 dpi but was not observed for F1 treatment (**Table 5**). At 30 dpi, F2 remained significantly protective for both direct and systemic treatments at all concentrations compared to the water treatment (**Figure S5D**). At 30 dpi, F2 was protective at all concentrations (**Figure S5D**), while F1 and D3 were not (data not shown).

To analyze the temporal regulation of H_2_O_2_ production, D3 and the lower MW fractions were evaluated using a time course series (**Figure 6**). Unlike the water and D1 treated and mock inoculated leaflets, treatment with 0.5 mg/mL of F1 and F3 and 1 mg/mL of F2 + mock inoculation resulted in H_2_O_2_ accumulation that was indiscriminate across the leaflets at 20 hpi (**Figure 6B**). In contrast to D1 and D2 treatment, H_2_O_2_ accumulation was macroscopically visible from 16 hpi onwards in D3 treated + *B. cinerea* inoculated leaflets (**Figure 6C**). No discernible differences in the patterns of H_2_O_2_ accumulation for *B. cinerea* inoculated leaflets were observed for all concentrations of F1, F2, and F3 assessed (**Figure 6C**). However, these MW fractions exhibited H_2_O_2_ accumulation as early as 4 hpi compared to 16 hpi for D3 treated + *B. cinerea* inoculated leaflets (**Figure 6C**). Furthermore, an increase in concentration resulted in a marginal decrease in the intensity of DAB staining for D3, F1, F2, and F3 as evident from the differences in the quantity and color intensity of brown lesion spots (**Figures 6A, C**).

### 3.4 The role of aminochitosan in priming *ACRE75*

De Vega et al. (2021) investigated and reported induced resistance and the temporal priming of Avr9/Cf-9 rapidly elicited protein 75 (*ACRE75*) by a commercially available water-soluble chitosan in tomato leaf discs infected with *B. cinerea* (De Vega et al., 2021). In the present study, the relative expression levels of *ACRE75* were measured in response to mock/*B. cinerea* inoculation and different concentrations of direct D3 treatment at 6 and 9 hpi and systemic D3 treatment at 96 hpi.

**Figure 7:**
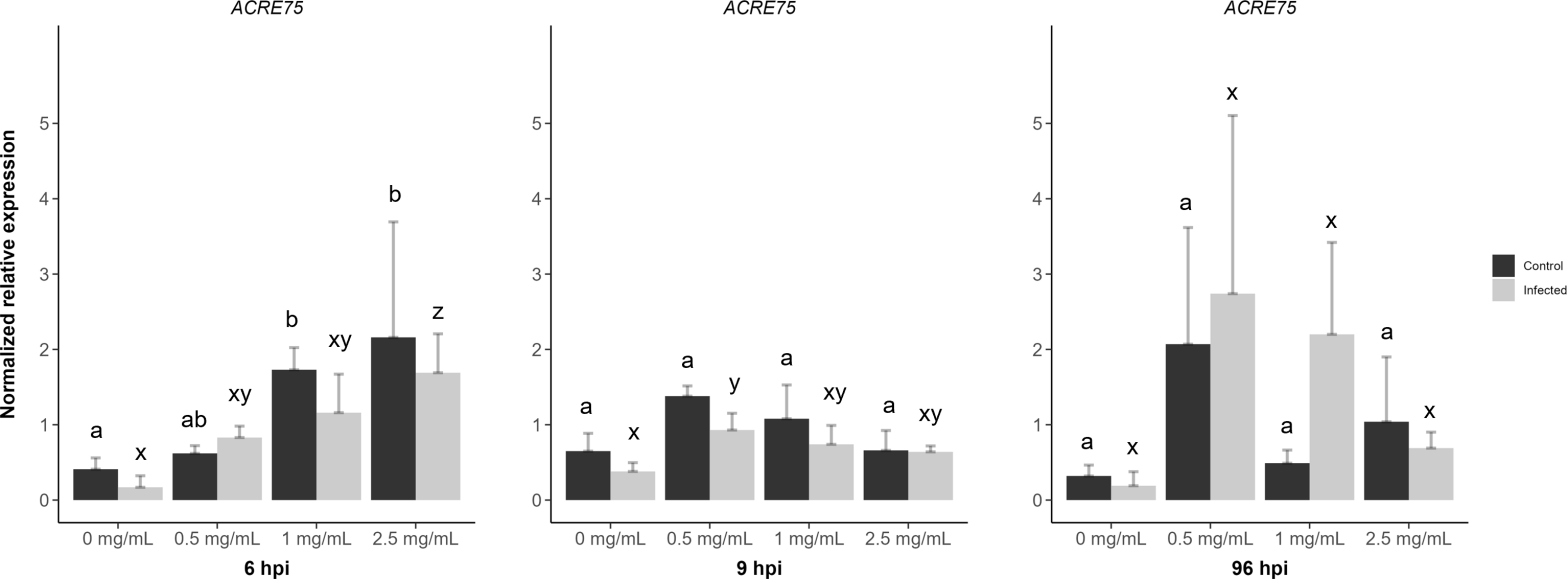
The effects of diamino 3 (D3) treatment compared to water treatment in *B. cinerea* and mock inoculated leaflets on the priming of *ACRE75* at 6, 9 and 96 hours post inoculation (hpi). Different letters indicate statistically significant differences. a/b indicate differences between controls and x/y/z indicate differences between infected samples. (Kruskal Wallis test followed by Dunn’s *post-hoc* test, p< 0.05). n = 3 per concentration.

D3 treated + mock inoculated leaflets displayed a significant increase in *ACRE75* normalized expression levels for 1 and 2.5 mg/mL concentrations at 6 hpi when compared to the water treatment (**Figure 7**). However, D3 treated + *B. cinerea* inoculated leaflets at 6 hpi had lower *ACRE75* relative expression levels compared to mock inoculated leaflets (**Figure 7**). Treatment with 2.5 mg/mL of D3 was significantly different from the water treatment at 6 hpi in *B. cinerea* inoculated leaflets. The 2.5 mg/mL concentration also showed the highest average relative expression levels for *ACRE75* at 6 hpi in D3 treated + mock/*B. cinerea* inoculated leaflets (**Figure 7**). Thus, at 6 hours post-inoculation, both the mock and *B. cinerea* inoculated leaflets exhibited a concentration-dependent increase in normalized relative gene expression **(** **Figure 7**).

However, at 9 hpi, the relative gene expression decreased with increasing concentration for both genes, with 0.5 mg/mL of D3 treatment showing the highest nonsignificant averages for both mock and *B. cinerea* inoculated leaflets (**Figure 7**). A similar trend was observed with systemic D3 treatment and + mock/*B. cinerea* inoculation at 96 hpi (**Figure 7**). Overall, *ACRE75* had higher average expression levels in the D3 treated and mock inoculated leaflets compared to the water treatment at both 6 and 9 hpi (**Figure 7**). However, the standard deviation for D3 treatments was greater, specifically in the treated and *B. cinerea* inoculated leaflets compared to the mock inoculated leaflets (**Figure 7**).

## 4 Discussion

Considering that amino groups are a defining factor in the physiochemical and biological properties of CHT derivatives; aminochitosan possesses improved functionality compared to native CHT (Satoh et al., 2006; Yang et al., 2012; Yang et al., 2015; Luan et al., 2018). To date, this is the first study to characterize aminochitosan dissolved exclusively in neutral water (pH = 7) for its antifungal properties *in vitro* and *in vivo*, with a focus on the tomato*/B. cinerea* pathosystem. However, batch-to-batch variations posed a challenge in obtaining consistent data in characterization of physiochemical properties and biological activity. One such variation noted was in the solubility of D2 which was not observed for D1, D3, or the lower MW fractions of D3. However, when comparing the elemental composition of the batches, minor variation was seen between D1 and D2 with a bigger difference being seen in D3 (with no effect on the solubility). Thus, these differences are potentially due to the variability in the source materials utilized to synthesize aminochitosan batches (Croisier and Jérôme, 2013; Sayed, 2018). Other known contributors to the solubility of CHT and derivatives are DDA, DS and MW (Luan et al., 2018). The DDA and DS determine the number of amine groups available for protonation, while the MW affects the number of charged amino groups available for intermolecular interactions with the solvent (water) (Croisier and Jérôme, 2013; Bellich et al., 2016). Aminochitosan with a high DDA and DS and a low MW is more soluble than aminochitosan with a low DDA, DS and a high MW (Bellich et al., 2016). Therefore, potential differences in MW resulted in small but measurable variations in the efficacy of the batches both *in vitro* and *in vivo*, as observed between D1 and D2.

### 4.1 Aminochitosan *in vitro*

Aminochitosan, specifically D1, displayed significantly greater inhibition of *B. cinerea* than CHT *in vitro* at a minimum concentration of 1 mg/mL. Similar results were reported by De Vega et al. (2021), who reported significant *in vitro* inhibition of *B. cinerea* with water-soluble CHT at a concentration of 0.1% (1 mg/mL) and higher (De Vega et al., 2021). Another study on aminochitosan (DS of 0.81, C/N 2.834) and CHT (DDA of 95% and MW of 700 kDa) by Luan et al. (2018) was investigated against various species in the *Fusarium* genus. The study showed improved antifungal activity greater than 20% for aminochitosan at 0.5 mg/mL. However, both CHT and their aminochitosan were dissolved in 0.35% acetic acid (Luan et al., 2018). In the present study, the *in vitro* antifungal activity of CHT was confounded using acetic acid as a solvent for CHT on account of acetic acid displaying significant radial growth inhibition *in vitro* at a concentration of 0.1%. Furthermore, these results were no different from the effects of the 0.5 and 1 mg/mL concentrations of CHT, indicating a protective effect of 0.1% acetic acid. Acetic acid, amongst others, has been shown to display antimicrobial activity *in vivo* and *in vitro* as the undissociated form of acetic acid is lipophilic, allowing penetration of the cell membrane (Narendranath et al, 2001). Once inside the cell, a decrease in the pH of the cytoplasm disrupts the cell membrane and inhibits metabolic processes necessary for fungal growth (Kang et al, 2003; Hassan et al., 2012; In et al., 2013). A study by Narendranath, Thomas & Ingledew (2001) reported a reduction in growth rates and glucose consumption of *S. cerevisiae in vitro* as the concentration of acetic acid in the media increased (Narendranath et al, 2001).

In the present study, a concentration-dependent increase in the *in vitro* antifungal activities of CHT, D1, and D2 was observed for radial growth and sporulation compared to the PDA control. Maximum inhibition for all the biopolymers was seen between the 2.5 and 5 mg/mL concentrations. Large standard deviations between the biopolymers for these concentrations were potentially due to the abovementioned batch-to-batch variations and inhibitory effects of acetic acid. At 0.5 mg/mL, an increase in sporulation and the number of spores/mL was observed for CHT, D2, and D1 in that respective biopolymer order. This could be attributed to the differences in the DDA and DS between CHT and aminochitosan. At low concentrations, CHT is the least inhibitory as it has a lower DDA than aminochitosan, whereas D1 and D2 have greater DDA and DS. However, no general trend can be deduced from the increase or decrease in DDA (Younes et al., 2014).

In addition, the haloes observed around the mycelial growth for CHT are indicative of the ability of *B. cinerea* to degrade and release CHT into the media thereafter. A study by Palma-Guerrero et al. (2007) reported a similar result for *Verticillium dahlia*, where its growth on PDA increased at 0.5 and 1 mg/mL and only decreased at 2 mg/mL. They suggested that *V. dahlia* was capable of using CHT as a nutrient source at lower concentrations and also reported the degradation activity of CHT, as noted by the appearance of halos around the mycelial growth at 0.5 and 1 mg/mL concentrations (Palma-Guerrero et al., 2007). Therefore, at low concentrations, *B. cinerea* may utilize aminochitosan as a nutrient source, with an apparent preference for an increased number of amine groups. This was noted in the differences between D1 and D2 inhibition, where D1 displayed the greatest increase in sporulation, which additionally correlated with its higher nitrogen percentage due to extra amine group(s) on aminochitosan. Harper et al. (1981) showed that omitting NH_4_NO_2_ from the growth medium resulted in a significant decrease in the *in vitro* growth of *B. cinerea* and in the percentage of spreading lesions *in vivo*. They concluded that nitrogen sources such as nitrate or ammonium support and enhance *in vitro* growth (Harper et al, 1981).

The germination data for D1 suggests the presence of a concentration threshold beyond which impaired germination or complete inhibition of germination occurs for aminochitosan and is maintained over time. The data in this study suggests that this threshold for aminochitosan is 1 mg/mL for spores germinating *in vivo*, compared to the 2.5 mg/mL threshold *in vitro*. Similarly, a study by Palma-Guerrero et al. (2007) reported that the spores of two plant pathogenic and two myco-parasitic fungi were more sensitive to CHT treatment than hyphae as growth was irreversibly inhibited at a concentration of 0.01 mg/mL (Palma-Guerrero et al., 2007). Hence, the *B. cinerea* spore suspension used *in vivo* was more sensitive than the *B. cinerea* fungal discs used *in vitro*, which contained a mixture of hyphae and spores. The contrast between *in vitro* and *in vivo* efficacy highlights the bimodal MOA of aminochitosan and the advantageous role of primed plant defense systems.

### 4.2 Aminochitosan *in vivo*

Image-based quantification of photosynthetic parameters is non-destructive, non-invasive, sensitive, rapid, and allows for high-throughput screening (Meng et al., 2020; Pavicic et al., 2021). Chlorophyll fluorescence imaging is generally used to assess and quantify the photosynthetic performance and efficiency of leaves including plant-pathogen interactions (Rolfe & Scholes, 2010; Pérez-Bueno et al, 2019). Furthermore, it accounts for the spatiotemporal heterogeneity of photosynthesis across the total leaf area (Bayçu et al., 2018). Plant-pathogen interactions regularly result in altered energy expenditure as a defense strategy and a decrease in photosynthesis and related chloroplastic metabolisms after the onset of chlorosis and necrosis at local infection sites (Berger et al., 2007; Fagard et al., 2014; Rojas et al., 2014). In order to analyze maximum photosynthetic efficiency of Photosystem II (PSII, also a measure of F_v_/F_m_), it is necessary to distinguish between the rates of photosynthesis, fluorescence emission, and heat dissipation as these factors are in competition with each other (Murchie & Lawson, 2013; Pérez-Bueno et al, 2019). When challenged, plants adapt by increasing their capacity for heat dissipation, while F_v_/F_m_ remains unchanged. However, if the stressor exceeds this adaptive capacity, a decrease in F_v_/F_m_ is observed, with the potential for extreme inhibition of PSII activity (Pérez-Bueno et al, 2019).

Image-based analysis corroborated the RGB findings (aminochitosan as a protective treatment up to 4 and 6 dpi) by analyzing photosynthetic parameters (F_v_/F_m_, ChlIdx, and mAriIdx). F_v_/F_m_ was shown to be inversely associated with lesion development as noted by the absence of “red lesions” with an increase in F_v_/F_m_ or by an increase in lesion size and disease progression with decreasing F_v_/F_m_ (Rolfe & Scholes, 2010; Meng et al., 2020). F_v_/F_m_ is therefore a useful indicator for the early signs of priming, infection, locally enhanced photosynthesis and a potentially enhanced defense response as a means of constraining pathogen growth to the site of infection (Berger et al., 2004; Rolfe & Scholes, 2010; Meng et al., 2020).

The data in the present study show enhanced photosynthesis in both the inoculation droplet site and in the surrounding areas. However, this observation is not restricted to the intercostal areas containing infection sites, as observed in Berger et al. (2004); rather, it is ubiquitous across the lamina. Therefore, maintaining heterogenous photosynthesis for as long as possible is a key aspect of the plant’s defense strategy (Berger et al., 2004). The sustained elevated photosynthetic activity may be due to priming of a stronger and more rapid elicitation of the defense systems at earlier time points, resulting in an unsuccessful infection. This is in contrast to the various chlorophyll fluorescence imaging studies on a few pathosystems, including the tomato/*B. cinerea* pathosystem, that have shown the downregulation of photosynthesis, chlorophyll fluorescence, and induction of sink metabolism after compatible pathogen interactions locally at the site of interaction and in surrounding tissues (Berger et al., 2004; Scharte et al, 2005; Bonfig et al., 2006; Berger et al, 2007; Muniz et al., 2014; Smith et al., 2014; Meng et al., 2020). As stated in Kanwar & Jha (2019), the data from Chou et al. (2000) and Berger et al. (2004) suggest that necrotrophic interactions generally result in rapid changes to photosynthesis that are visible before any apparent disease phenotype (Kanwar and Jha, 2019). The D1 data agree with this observation, where changes in photosynthesis are sustained and quantified, extending up to 4 and 6 dpi without an apparent disease phenotype. However, the D2 data is similar to that of Chou et al. (2000) and Berger et al. (2004), where infected leaves generally displayed inhibition of photosynthesis at the site of infection with an area of maintained photosynthetic parameters (healthy areas) in the immediate surrounding uninfected leaf areas, noted as “green islands”, is a representation of the spatiotemporal heterogeneity of infection (Chou et al., 2000; Berger et al., 2004; Pérez-Bueno et al, 2019).

The term “green island” has been a descriptor for biotrophic interactions where areas of senescence are halted and photosynthetic activity is maintained, although at a lower level. Therefore, the occurrence of green islands is generally seen at later stages of disease progression, where the site of infection remains green while the surrounding tissue senesces (Walters et al, 2007). Polyamines (PA) are a group of compounds that retard senescence and accumulate in green islands (Walters et al, 2007). Naturally occurring PA, such as spermine and spermidine, are synthesized in plants and are defined as low molecular weight polycations containing amino groups (Janse van Rensburg et al, 2021). PAs are both water-soluble and insoluble as they exist in “free” or conjugated forms (Janse van Rensburg et al, 2021). Not dissimilar to aminochitosan (and in contrast to CHT), this property coupled with its positive charge allows for differential distribution and localization as well as electrostatic interactions with nucleic acids, acidic proteins, and phospholipids (Janse van Rensburg et al, 2021). Aminochitosan therefore bears similarity to PAs; its biological activity may be mediated through similar mechanisms and pathways that prevent senescence, resulting in a resistant phenotype of varying degrees. Comparably, exogenous PA application was shown to prime resistance and increase stress tolerance to *B. cinerea* infection in Arabidopsis (Janse van Rensburg et al, 2021), maintain the integrity of the thylakoid membrane during leaf senescence (Besford et al., 1993), prevent the loss of or elevate chlorophyll content (Galston & Sawhney, 1990; ElSayed et al., 2022), maintain normal or elevated PSII activity (Legocka and Zajchert, 1999; ElSayed et al., 2022), and impede the initial stages of crown rust infection by affecting germ tube growth and appressorium formation (Montilla-Bascón et al., 2016). Furthermore, high total chlorophyll was correlated with basal leaf resistance (Meng et al, 2019). In this study, foliar application of aminochitosan displayed efficacy in priming direct resistance to *B. cinerea* infection by maintaining elevated ChlIdx and PSII activity as well as directly inhibiting germination *in vivo*.

Interestingly, a decrease in F_v_/F_m_, and therefore photosynthesis, was observed at 2.5 mg/mL of aminochitosan application. This may be indicative of a decrease in the efficiency of PSII due to the destabilization of chloroplasts and thus PSII (Meng et al., 2020). Hence, at 2.5 mg/mL, aminochitosan may be moderately cytotoxic when sprayed directly onto leaves. The decrease in F_v_/F_m_ visually and quantitatively overlapped with the decrease in ChlIdx at 2.5 mg/mL as areas with residual dry droplets matched areas of decreased ChlIdx. This observation was also noted for the mock inoculated leaves treated with 2.5 mg/mL of D1 and therefore indicates that the observed effects are not due to the establishment of an infection but rather to the concentration of the treatment. Moreover, this appears to be concentration-dependent, as the same observation is absent at 0.5 mg/mL but can be seen for several leaves at 1 mg/mL. Similarly, various studies have reported negative effects on the establishment of necrotic lesions and their severity with high concentrations of exogenous PA application or endogenous accumulation (Yoda et al, 2003; Marina et al., 2008; Nambeesan et al., 2012).

In this study, in addition to F_v_/F_m_ and ChlIdx, mAriIdx was used as a measure of anthocyanin accumulation in leaves (Meng et al., 2020). Anthocyanins are reported to have putative functions in halting leaf senescence as well as being regulators of ROS signaling pathways (Hatier and Gould, 2008). The accumulation of anthocyanin was visible at the *B. cinerea* inoculation sites in water treated leaflets but was variable in aminochitosan treated leaflets. Similar results were observed by Meng et al. (2020), where mAriIdx was seen accumulating at the site of infection with *B. cinerea* in untreated leaves (Meng et al., 2020). In the present study, the accumulation of anthocyanins appeared to visually decrease with an increase in the concentration of aminochitosan at the site of *B. cinerea* inoculation. This suggests that anthocyanin accumulation is an indicator of leaf susceptibility to successful infections when treated with aminochitosan. Leaves treated with 0.5 mg/mL of aminochitosan had greater anthocyanin accumulation and disease resistance than the water treatment. However, they were more susceptible than those treated with 1 and 2.5 mg/mL where little to no anthocyanins were visible at the sites of inoculation thus indicating a resistant interaction. This suggests priming mechanisms that are independent of anthocyanin accumulation and ROS accumulation at later time points when treated with higher concentrations of aminochitosan in *B. cinerea* inoculated leaves. A likely explanation is that the direct antifungal activity of aminochitosan at higher concentrations is severe, resulting in lower ROS production, less oxidative stress (HR-like response), and lower anthocyanin concentrations than at lower concentrations of aminochitosan.

In the mock inoculated leaves treated with aminochitosan at 1 and 2.5 mg/mL, anthocyanin accumulation overlapped with the dried aminochitosan droplets. In contrast to infected leaves, this suggests that foliar anthocyanins are primed in uninfected leaves in response to higher concentrations of aminochitosan. Additionally, the enhanced F_v_/F_m_ values and thereby enhanced photosynthetic activity observed in these leaves may indicate an increase in starch and sugar production. As sugar accumulation is positively correlated with anthocyanin concentration, accumulation in older leaves may act as a mechanism for regulating sugar content in an attempt to circumvent early senescence elicited by high sugar levels in source tissues (Pourtau et al., 2006; Landi et al, 2015). Thus, anthocyanins are potentially alternative sinks that avoid excess carbon and sugar accumulation to mitigate possible “sugar-induced leaf senescence” induced by enhanced photosynthetic activity after application of a high concentration of aminochitosan (Landi et al, 2015).

In addition to the local resistance induced by direct CHT application, systemic resistance has also been reported for various pathosystems (Benhamou & Thériault, 1992; Vasyukova et al., 2001; Faoro et al., 2008; Siddaiah et al., 2018). The significantly elevated F_v_/F_m_ induced by direct aminochitosan application was also seen with the systemic pre-treatment of D1 at 4 and 6 dpi. This corresponded to a reduction in lesion sizes and the number of spreading lesions. The effects were concentration-dependent, with 1 and 2.5 mg/mL performing significantly better than 0.5 mg/mL but still being protective at 0.5 mg/mL. Notably, at both 0.5 mg/mL and 1 mg/mL, the occurrence of spreading and resistant lesions on individual leaflets varied, as regulation of the defense systems is expectedly heterogeneous within each individual leaflet (Pérez-Bueno et al, 2019). The successful priming of a resistant response systemically highlights the benefits of a more efficient and effective induction of the innate immune system globally (Pastor et al., 2013).

Priming results in a combination of physical and chemical responses that include ROS and have been reported for a variety of pathosystems using CHT and derivatives at various concentrations and stages of development (Rabea et al., 2003; Raafat et al., 2008; Goy et al, 2009; Iriti & Faoro, 2009; Hadwiger, 2013). H_2_O_2_ accumulation is a crucial, early-phase defense response that functions as a signaling molecule, a cell wall modifier, and a mediator of hypersensitive responses (Lin et al., 2005; Asselbergh et al., 2007). In this study, lesions were absent at 1 and 2.5 mg/mL of D1 application, as in the whole leaf analysis, and was coupled with generally little to no H_2_O_2_ accumulation at these concentrations (a decrease in accumulation with an increase in D1 concentration). Additionally, the time taken to accumulate H_2_O_2_ comparable to the water treatment increased with an increase in concentration. These results corroborate the aforementioned anthocyanin and direct *in vitro* data that suggest that aminochitosan functions in a ROS-independent manner, especially at higher concentrations where direct inhibition takes precedence.

Despite H_2_O_2_ generally being a marker for an upregulated defense response, it is also known to contribute to successful infections by necrotrophs such as *B. cinerea* (Stamelou et al., 2021). Meng et al. (2019) reported that in strawberry leaves infected with *B. cinerea*, “H_2_O_2_ levels were positively correlated with disease severity” and that lower levels were a better indicator for resistance (Meng et al, 2019). Other studies have reported similar effects of high H_2_O_2_ levels and hypersensitive responses having a positive correlation with necrosis (Govrin & Levine, 2000; Khanam et al., 2005). The effects on PSII functionality reported by Adamakis et al. (2020) and Stamelou et al. (2021), showed that lower ROS levels was favorable for the activation of defense responses, whereas high ROS levels were detrimental to the functionality of PSII, indicating toxicity (Adamakis et al., 2020; Stamelou et al., 2021). Hence, lower concentrations of aminochitosan may be favoured due to its low and slow increase in H_2_O_2_ levels, resulting in maintained PSII functionality compared to the decrease seen at 2.5 mg/mL. Adamakis et al. (2020) and Stamelou et al. (2021) suggested that with short-term exposure, PSII functionality increased rapidly but that with longer exposure, inhibition indicated a “time-dependent hormetic response”. Hormesis typically denotes a biphasic response that is depicted by a U/J shape to a stress or elicitor that elicits advantageous effects at low concentrations (eustress) and a toxic effect at high concentrations (Tran et al., 2011). Therefore, at time points earlier than 4 dpi, 2.5 mg/mL of aminochitosan is beneficial to the leaves, but at later time points, the benefits decrease. A functional use of this dose response in plants is for elucidating optimal biostimulant concentrations that achieve the best adaptive response to disease resistance (Tran et al., 2011).

The microscopic observations also revealed an interaction between D1 and Trypan Blue, which can be seen by the appearance of blue circles correlating with the droplet residues that remained on the leaf tissue after treatment with D1. As Trypan Blue is a negatively charged diazo dye, it is capable of interacting with a cationic compound such as aminochitosan (Tran et al., 2011), or in this instance, D1. Therefore, Trypan Blue permeates through the cell walls of living cells that have altered membrane permeability due to the interaction with aminochitosan (Tran et al., 2011). As stated in Tran et al. (2011), the blue coloration of cells should be assessed with caution as it may not signify cell lysis but rather an increase in membrane permeability due to pore formation (Tran et al., 2011). Therefore, this suggests that aminochitosan acts to increase the membrane permeability of cells after foliar spray thereby allowing the permeation of aminochitosan into the cell membranes and cells. Most notably, when compared to the spores outside of the D1 droplet area, the spores beneath the D1 droplet area had minimal or no germination efficacy. In addition to destabilizing the cell membrane, the film-forming properties of CHT may function as a physical barrier to the efflux of nutrients from the plant, thereby reducing nutrient availability for fungal growth. This theory has been supported by studies that show nutrient deprivation and a lack of fungal growth as a result of these film-forming properties (El-Ghaouth et al., 2000; Ait Barka et al., 2004). These observations corroborate the notion that aminochitosan biopolymers like D1 can exert their effects through both direct MOA and indirect immunostimulatory mechanisms.

### 4.3 The importance of molecular weight on the biological activity of aminochitosan: finding the optimal balance between *in vitro* and *in vivo* efficacy

The D3 MW fractions were analyzed to assist in determining the optimal MW range for future applications of aminochitosan in the tomato/*B. cinerea* pathosystem and others. The elemental analysis (EA) results verified the higher DDA in aminochitosan, as evidenced by the elevated percentages of nitrogen compared to CHT. As per the literature, the C/N ratios of aminochitosan and fractions in this study were closer to that of completely deacetylated chitosan (5.145) compared to CHT which was closer to chitin (6.861), the completely N-acetylated biopolymer (Galed et al., 2008). The proportion of nitrogen between the biopolymers differed slightly, with the amino biopolymers exceeding the value for CHT. These values are in agreement with the DS values reported in studies on aminochitosan and range between 0.70-0.98 (Satoh et al., 2006; Yang et al., 2012; Luan et al., 2018; Sayed, 2018). Therefore, it may be assumed that the differences in efficacy between the biopolymers are not due to their elemental composition and proportions and are potentially due to MW differences. Although, it is worth noting that EA has certain limitations that may lead to an overestimation of the DS (Sayed, 2018).

However, despite the relatively high DS values and elevated nitrogen percentages for the D3 MW fractions, the antifungal activity of the biopolymers *in vitro* was variable relative to the EA data. In summary, the results demonstrated that a minimum concentration of 1 mg/mL is required for significant direct antifungal activity of D3 and D3 MW fractions. Furthermore, an increase in the efficacy of the concentrations with a decrease in the MW was observed, indicating a trend between the MW and biological activity. From this study, the MW range of 3.5-15 kDa (F1) appeared to be the most effective for *in vitro* activity against *B. cinerea* at concentrations of 0.5, 1, and 2.5 mg/mL. However, it is worth noting that the differences in MW efficacy may be influenced by the potential agglomerative nature of CHT in the culture medium. This implies that the formation of aggregates between CHT and the media decreases the theoretical amount of chitosan dissolved in solution and impedes *in vitro* antifungal activity (Lee et al, 2016). Nonetheless, the findings of this study agree with the results of Hernández-Lauzardo et al. (2008) and Badawy and Rabea (2009). They showed that low MW CHT had stronger antifungal effects against *Rhizopus stolonifera* and *B. cinerea* compared to high MW CHT at a concentration between 0.5 mg/mL and 4 mg/mL (Hernández-Lauzardo et al., 2008; Badawy & Rabea, 2009). In these studies, a wide range of MW values were defined, with Hernández-Lauzardo et al. (2008) defining low to high MW as 17.4-30.7 kDa and Badawy and Rabea (2009) defining their low to high MW range from 5-57 kDa, with an ultra-high MW of 290 kDa. The proposed variations in the mode of action of CHT and aminochitosan are consistent with both low and high MW. Low MW CHT disrupts the fungal cell wall more efficiently due to its smaller size (as with F1), while high MW CHT creates a protective barrier or film that reduces microbial growth by binding to the cell surface (as evident from the reduced antifungal efficacy of the F4 (20-99 kDa) and F5 (100 kDa) (Bellich et al., 2016).

In the present study, the *in vitro* MW range for optimal biological activity of 3.5-15 kDa is in contrast with the optimal *in vivo* MW range of 15-20 kDa. F1 was significantly protective, but only at the higher concentrations and was less protective than F2 (15 kDa) and F3 (20 kDa). These results are in opposition to findings reported by Vasyukova et al. (2001), who showed that maximal disease resistance to *Phytophthora infectans* infection in potatoes was displayed by low MW CHT (5 kDa) treatment compared to the intermediate effects of 24 kDa CHT and the ineffective 200 kDa CHT (Vasyukova et al., 2001). However, the variable definitions for MW ranges result in inconsistencies within the literature. The direct and systemic treatment with D3 and the lower MW fractions, F2 and F3, resulted in significant resistance at 4 and 6 dpi with F2 and F3 providing greater protection than D3. Notably, F2 and F3 displayed a decrease in resistance at 2.5 mg/mL, at 4 and 6 dpi when compared to 1 mg/mL of the respective biopolymers for direct and systemic treatment. These results correspond with the D1 *in vivo* results at 2.5 mg/mL, where a concentration-dependent threshold and response were observed. The disease resistance for direct and systemic treatment by F2 and F3 was also retained up to 30 dpi compared to D3, F1, and water treatment, which were not protective. This suggests that fractionating the biopolymer to a select MW results in stronger biological activity *in vivo* compared to a wider MW range found within a copolymer not strictly synthesized to a MW.

Consequently, it is noteworthy that the lower MW fractions showed no true discernible differences in the patterns of H_2_O_2_ accumulation for all concentrations of F1, F2, and F3 assessed. As hypothesized, F2 accumulation appeared closest to that of D3 as F2 and D3 are both approximately 15 kDa. Similar to D1 and D3 treated + *B. cinerea* inoculated leaflets, an increase in concentration resulted in a decrease in the intensity of DAB staining for F1, F2, and F3. Lin et al. (2005) demonstrated that CHT induced H_2_O_2_accumulation in rice cell culture; however, its capacity was dependent on the MW range of CHT (Lin et al., 2005). Like these findings, aminochitosan-induced H_2_O_2_accumulation in tomato leaflets inoculated with *B. cinerea* appeared to have variable time responses to the different MW fractions. This further highlights the importance of characterizing the optimal ranges of MW for maximum biological activity.

The impact of variations in MW on the antifungal activity of CHT and its derivatives is a widely studied topic with conflicting and inconclusive findings in the literature (Kong et al., 2010; Liaqat & Eltem, 2018; Poznanski et al, 2023). The absence of uniform definitions for “high” and “low” MW CHT in the literature adds to the inconsistencies since the ranges considered “high” and “low” overlap across studies and could therefore be defined oppositely in others. Moreover, the relationship between MW and antifungal activity is affected by intrinsic and extrinsic factors that include different organisms, ranges of MW, DDA, and DP, non-standardized concentrations, the source of chitin, and methods used to synthesize CHT. (Poznanski et al, 2023). Therefore, it is challenging to ascertain the ideal MW of aminochitosan to achieve maximum antifungal efficacy based on current data, and it should instead be chosen based on the intended application (Poznanski et al, 2023).

### 4.4 Aminochitosan primes *ACRE75*

ACRE genes have been reported as being involved in R gene-mediated defenses and various defense signaling pathways (Durrant et al., 2000). Furthermore, the induction of most ACRE genes may occur via ROS-independent pathways, as they do not require ROS for upregulation which may support the notion that aminochitosan primes a defense response in a ROS-independent manner or through late H_2_O_2_ accumulation (Durrant et al., 2000). Therefore, *ACRE75* was analyzed for temporal regulation in response to different concentrations of D3 treatment when applied as a direct treatment in mock or *B. cinerea* inoculated leaflets at 6, 9, or 96 hpi or as a systemic treatment in mock or *B. cinerea* inoculated leaflets at 96 hpi.

D3 treatment + mock inoculation resulted in greater average relative expression of *ACRE75* compared to the water treatment at both 6 and 9 hpi for 1 and 2.5 mg/mL. This observation suggests aminochitosan, an elicitor of *ACRE75* that is primed in both mock and *B. cinerea* inoculated leaflets for a stronger expression with and without infection. Moreover, the relative expression of *ACRE75* was positively regulated, with an increase in concentration at 6 hpi for both mock and *B. cinerea* infected leaflets, indicating a concentration-dependent response at the earlier time points. However, at 9 hpi, the greatest increase in relative expression was for 0.5 mg/mL, with a subsequent decrease in expression for 1 and 2.5 mg/mL for both mock + *B. cinerea* inoculated leaflets. A study by Iriti and Faoro (2009) noted concentration and the physiochemical properties of CHT as key factors modulating priming and direct defenses, as at certain concentrations, beneficial programmed cell death may switch to non-beneficial necrotic lesions due to cytotoxicity (Iriti & Faoro, 2009). Therefore, elucidating the optimal concentration for non-toxic priming is key to utilizing aminochitosan to its full potential. At 96 hpi, systemic treatment with 0.5 mg/mL displayed the greatest priming in both mock and *B. cinerea* inoculated leaflets. This suggests that lower concentrations of aminochitosan are sufficient for priming systemic accumulation of *ACRE75* and that the protection is sustained up to and including 96 hpi.

## 5 Conclusions

Here we have demonstrated aminochitosan as a preventative treatment to *B. cinerea* infection when applied as a foliar spray in 5-week-old tomato leaves. Aminochitosan displayed significantly improved biological activity *in vivo* when applied directly and as a systemic treatment that was sustained for up to 30 days post-inoculation. The resistant phenotype is mediated through a combination of enhanced F_v_/F_m_ and ChlIdx. The mechanism of action appears to be ROS-independent at higher concentrations due to the severity of direct inhibition. Consequently, leaf senescence, hypersensitive responses and therefore necrosis are mitigated which suggests that aminochitosan primes defense responses in both mock and *B. cinerea* inoculated leaves. However, the concentrations for optimal activity appear to differ *in vitro* and *in vivo*. Additionally, the lower MW fractions suggest a narrow range in which optimal/maximal efficacy is effected *in vitro* compared to *in vivo* which additionally is pathosystem dependent. This study provides a base for further research into the effects of aminochitosan in other pathosystems and larger field trials with a focus on “omics” for further elucidation on the MOA.

## Data availability statement

The datasets presented in this article are not readily available because they are part of doctoral thesis. Requests to access the datasets should be directed to suhail.rafudeen@uct.ac.za.

## Author contributions

NM: Formal Analysis, Methodology, Data curation, Investigation, Software, Validation, Visualization, Writing – original draft. AJ: Methodology, Resources, Supervision, Writing – review & editing. KA: Resources, Supervision, Writing – review & editing, Software. MR: Resources, Supervision, Writing – review & editing, Conceptualization, Formal Analysis, Funding acquisition, Methodology, Project administration.
