# Supplementary material for: 6-deoxy-6-amino chitosan: a preventative treatment in the tomato/Botrytis cinerea pathosystem
Source: Front Plant Sci. 2023 Oct 10;14:1282050. doi: 10.3389/fpls.2023.1282050 (PMC10595175; doi:10.3389/fpls.2023.1282050)
Supplement: Supplementary file 1 [file DataSheet_1.docx]

Supplementary Material

**Table S1**: The effects of the chitosan and aminochitosan variants on disease progression in *B. cinerea* inoculated leaf discs measured as the percentage of resistant lesions at 4, 8, 12, 16, 20, 24, 48 and 72 hpi.

| **Treatment** | **Concentration (mg/mL)** | **% Resistant lesions** | | | | | | | | |
| --- | --- | --- | --- | --- | --- | --- | --- | --- | --- | --- |
|  |  | **Time (hpi)** | | | | | | | | |
|  |  | **4** | **8** | **12** | **16** | **20** | **24** | **48** | **72** |  |
| Water | 0^a^ | 100^a^ | 100^a^ | 100^a^ | 13^a^ | 0^a^ | 0^a^ | 0^a^ | 0^a^ |  |
| 0.1% acetic acid | 0.1^ab^ | 100^a^ | 100^a^ | 100^a^ | 100^b^ | 0^a^ | 0^a^ | 0^a^ | 0^a^ |  |
| Chitosan | 0.5^ab^ | 100^a^ | 100^a^ | 100^a^ | 100^b^ | 38^ab^ | 13^a^ | 13^a^ | 0^a^ |  |
|  | 1^ab^ | 100^a^ | 100^a^ | 100^a^ | 100^b^ | 50^b^ | 25^a^ | 0^a^ | 0^a^ |  |
|  | 2.5^b^ | 100^a^ | 100^a^ | 100^a^ | 100^b^ | 88^b^ | 88^b^ | 25^a^ | 13^a^ |  |
| Water | 0^a^ | 100^a^ | 100^a^ | 100^a^ | 13^a^ | 0^a^ | 0^a^ | 0^a^ | 0^a^ |  |
| Diamino 1 | 0.5^b^ | 100^a^ | 100^a^ | 100^a^ | 100^b^ | 100^b^ | 63^b^ | 50^b^ | 25^a^ |  |
|  | 1^b^ | 100^a^ | 100^a^ | 100^a^ | 100^b^ | 100^b^ | 100^b^ | 75^b^ | 100^b^ |  |
|  | 2.5^c^ | 100^a^ | 100^a^ | 100^a^ | 100^b^ | 100^b^ | 100^b^ | 100^b^ | 100^b^ |  |

The data shows the average of two experiments with 5 leaflets per time point. The significance between concentrations for each polymer is denoted with letters; the same letters are not statistically different from each other (Kruskal Wallis test followed by Dunn’s post-hoc test, p < 0.05).


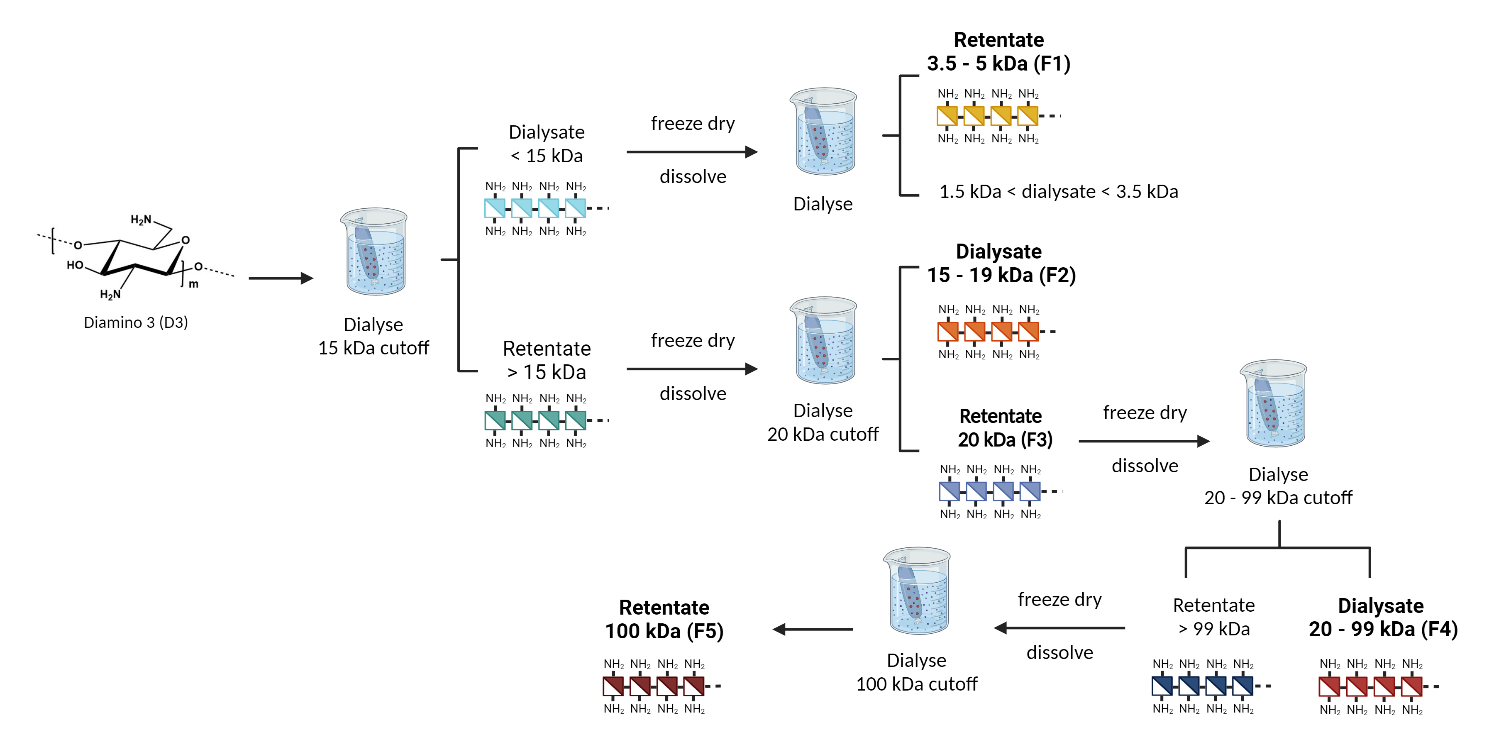


**Figure S1**: The fractionation process applied to obtain the diamino 3 (D3) MW fractions. The representative monomer structures are not drawn to true MW sizes. Created with BioRender.com.


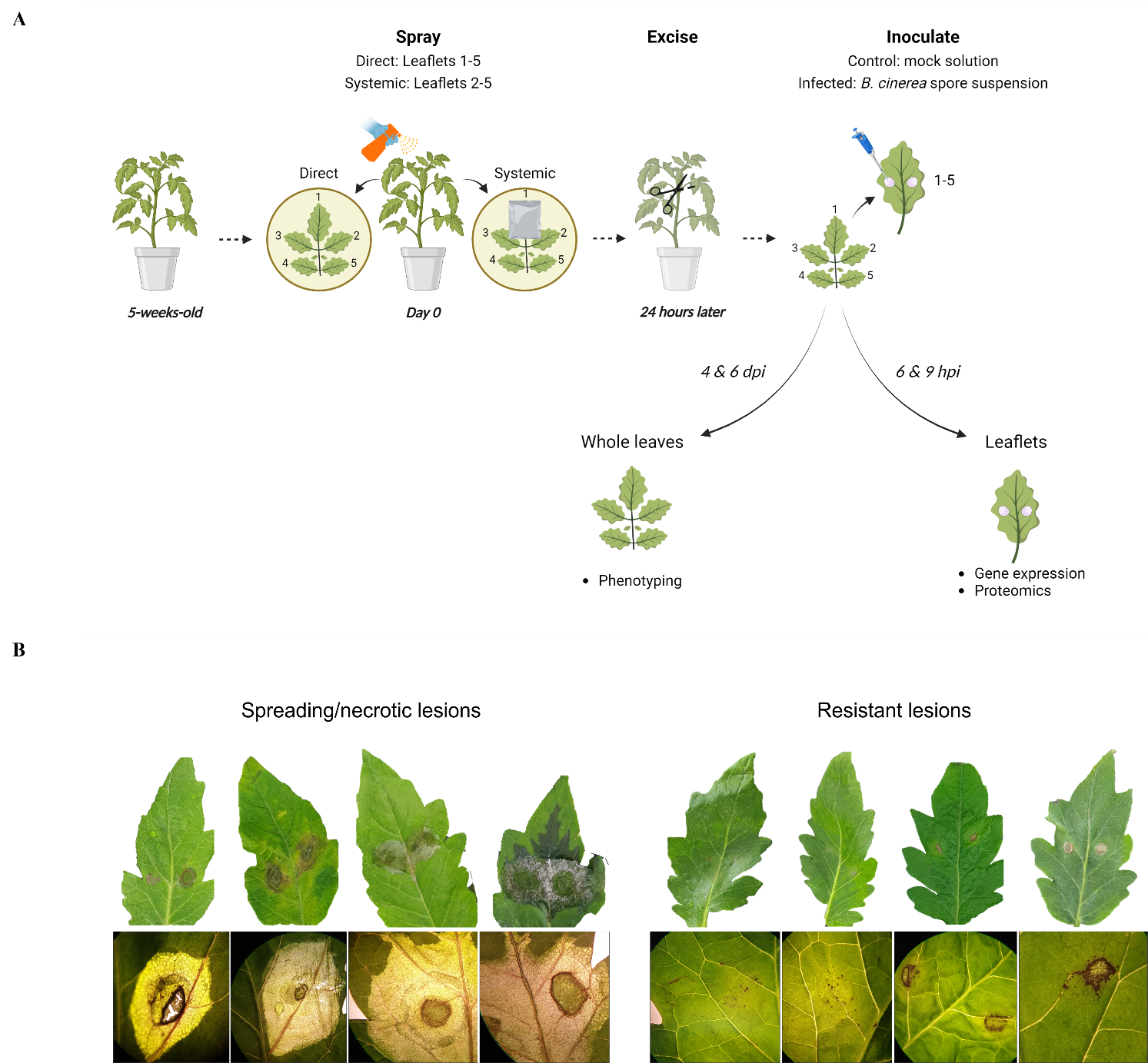


**Figure S2**: **(A)** Schematic of the experimental set-up used for the *in vivo* experiments. 5-week-old tomato plants were treated with a foliar spray of chitosan or aminochitosan at a concentration of 0.5, 1 or 2.5 mg/mL or control (water or 0.1% (v/v) acetic acid). After 24h, leaves were excised and inoculated with either a solution of *B. cinerea* spore suspension (1 x 10^6^ spores/mL containing 0.01 M glucose and 6.7 mM KH_2_PO_4_) or mock solution (water containing 0.01 M glucose and 6.7 mM KH_2_PO_4_). Leaves or leaflets were harvested at varying time points for phenotyping, gene expression or proteomics analysis. Disease progression is measured by the presence or absence of necrotic/resistant lesions. This figure was created with BioRender.com. **(B)** The range of phenotypical differences between spreading (susceptible) and resistant lesions. Spreading and necrotic lesions appear as a mixture of “wet”, light brown and with/without grey mycelia typically characterized by the initial appearance of primary dark brown necrotic spots around the edge of the lesion between 24 and 48 hpi. At 72 hpi the lesions vary in size with the defining trait of a “wet” or “water-soaked” lesion. Resistant lesions are lesions that do not progress to a “water-soaked” lesion and instead remain as dark brown spots in a small contained area. Resistant lesions may appear macroscopically as scatterings of brown or as white, dry, hardened, circular lesions.


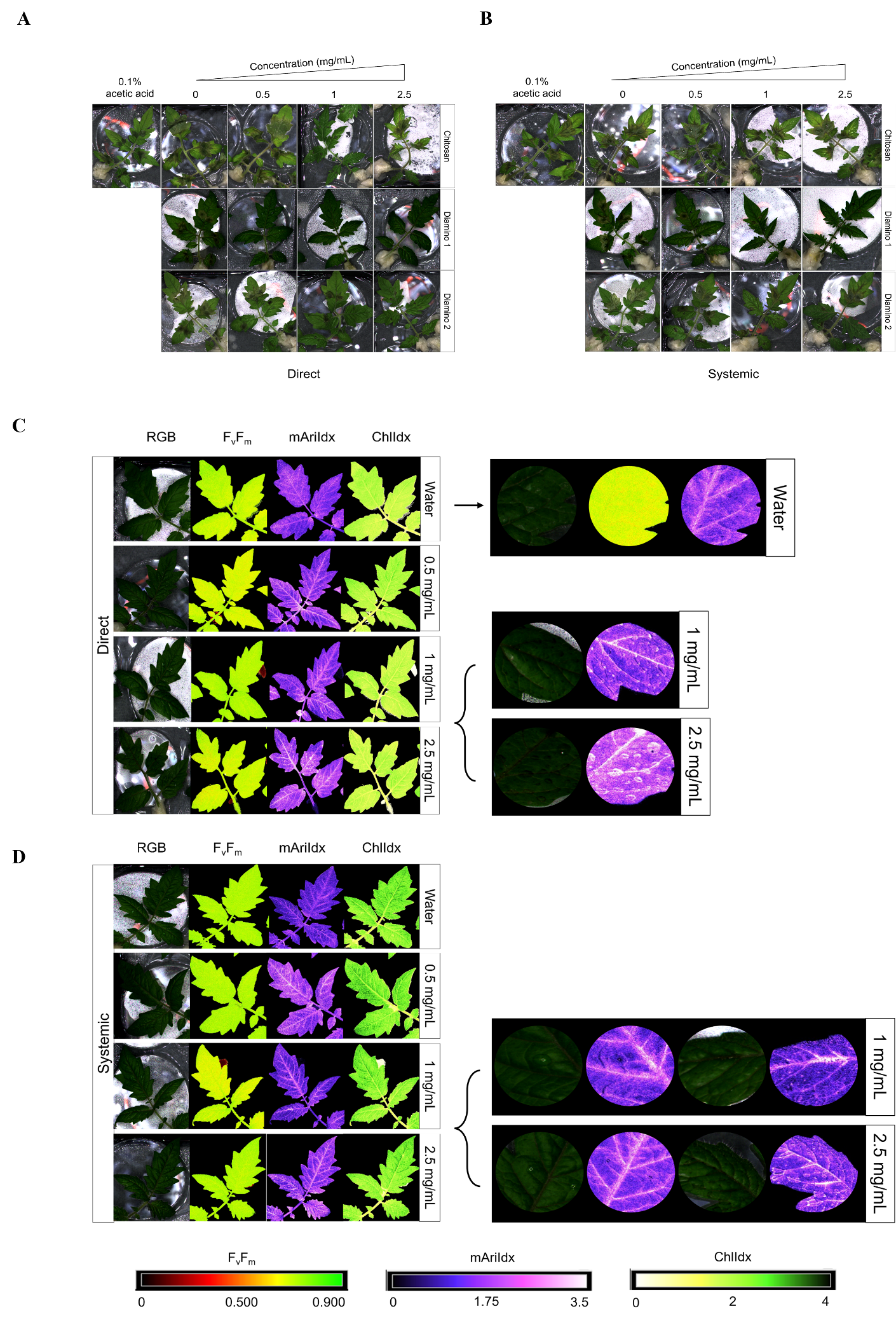
**Figure S3**: The effects of direct or systemic chitosan (CHT), diamino 1 (D1) and diamino 2 (D2) treatment on leaf health including photosynthetic parameters after *B. cinerea* inoculation at 4 days post inoculation (dpi). Lesion development and progression are noted by the spreading of dark red/black (F_v_/F_m_, 0-0.5) or yellow/white (ChlIdx, 0-1.8) spots as measured by the false color scales. Healthy leaf tissue is noted as yellow/green (F_v_/F_m_,0.7-0.9) or green (ChlIdx, 1.9-2.5) by the false color scales. **(A)** RGB images of leaves treated directly at 4 dpi. **(B)** RGB images of leaves treated systemically at 4 dpi. The images represent the average phenotype of 2 experiments. **(C)** RGB, F_v_/F_m_, AriIdx and ChlIdx images of direct D1 treatment in mock-inoculated leaflets at 4 dpi with a detailed view of the effects of direct treatment with 1 and 2.5 mg/mL. **(D)** RGB, F_v_/F_m_, AriIdx and ChlIdx images of systemic D1 treatment in mock-inoculated leaflets at 4 dpi with a detailed view of the effects of direct treatment with 1 and 2.5 mg/mL. Leaves were inoculated 24 hours after polymer spraying with two 10 µL droplets of a water solution containing 0.01 M glucose and 6.7 mM KH_2_PO_4_. The images represent the average phenotype of 2 experiments.


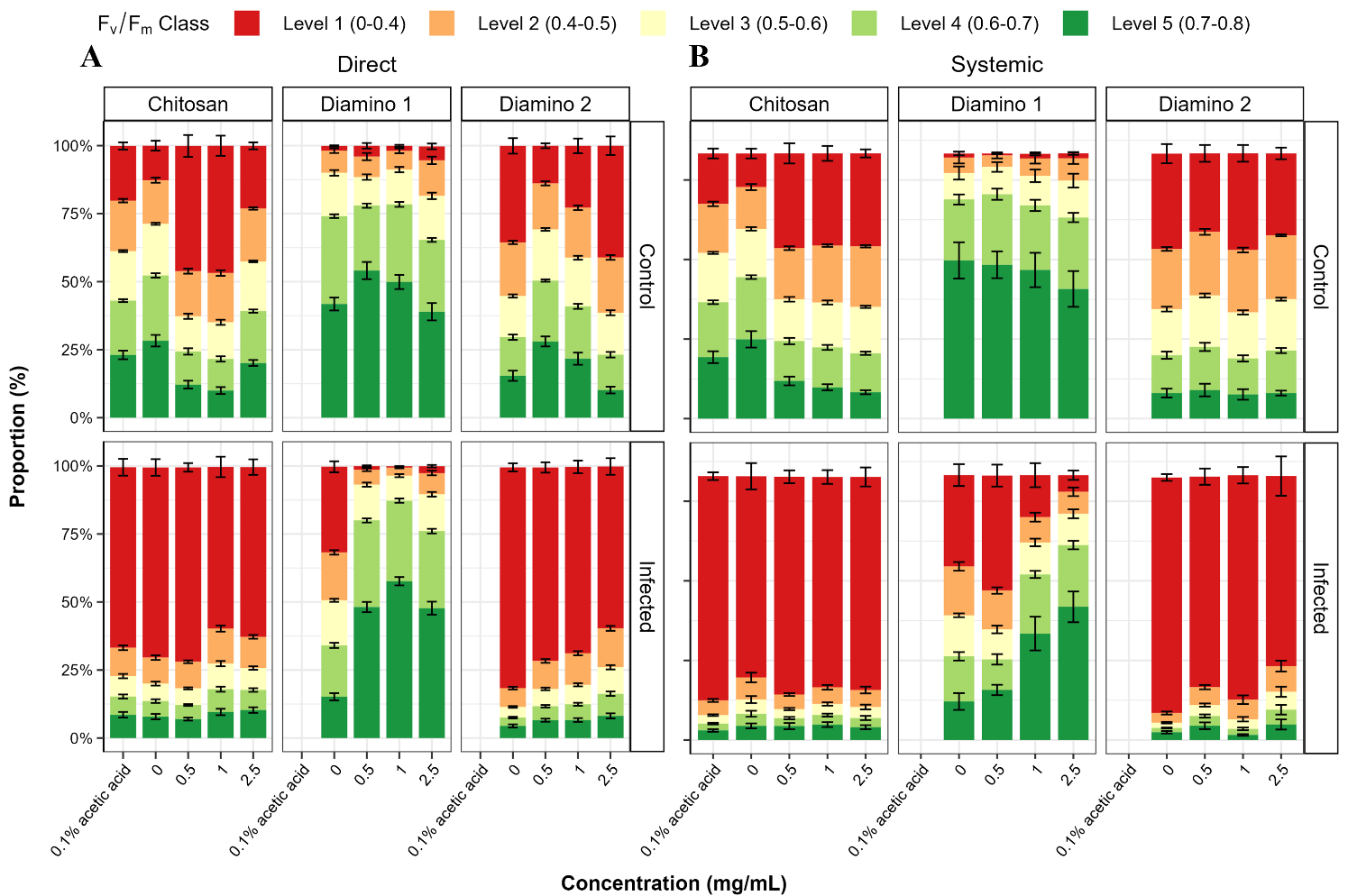
**Figure S4**: The effects of chitosan (CHT), diamino 1 (D1) and diamino 2 (D2) on the F_v_/F_m_ of mock and *B. cinerea* inoculated leaves, 4 days post-inoculation*.* Healthy leaf conditions are represented by levels 3, 4 and 5. Infected leaf conditions are represented by levels 1-2 with the highest severity of infection = level 1. n = 9 leaves, 45 leaflets per treatment.


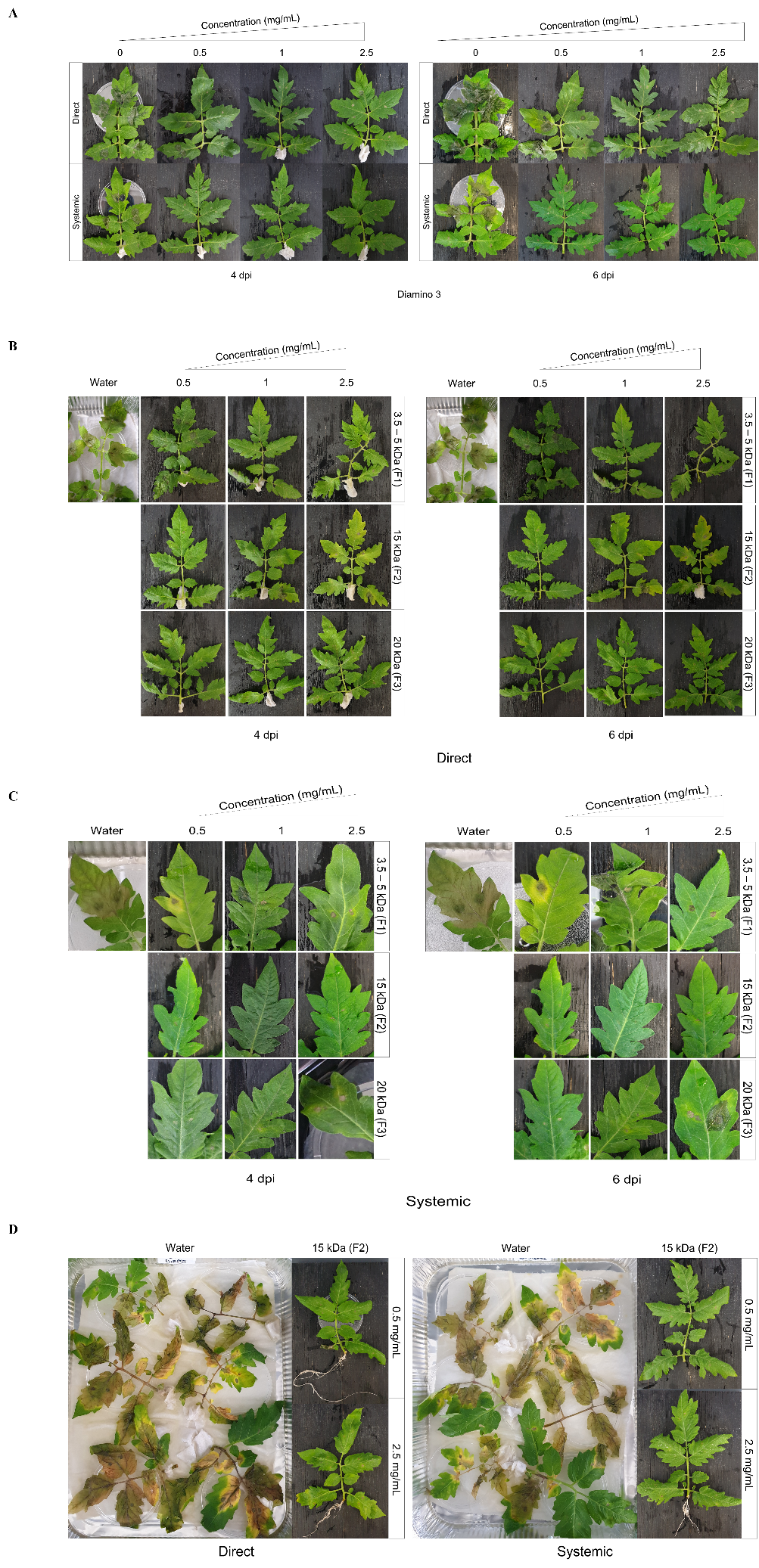


**Figure S5**: The phenotype of *B. cinerea* infected 5-week-old detached tomato leaves treated with various concentrations of diamino 3 (D3) and D3 MW fractions, at 4- and 6-days post inoculation (dpi). **(A)** Direct and systemic application of D3. (**B)** Direct effects of the MW fractions (F1-F3) **(C)** Systemic effects of the MW fractions (F1-F3). **(D)** Direct and systemic treatment of 0.5 and 2.5 mg/mL of F2 in *B. cinerea* inoculated leaves at 30 dpi. Leaves were inoculated with two 10 µL droplets of a *B. cinerea* spore solution (1 x 10^6^ spores/mL containing 0.01 M glucose and 6.7 mM KH_2_PO_4_). The images represent the average phenotype of 2 experimental repeats.
